# Supplementary material for: The CoDiNOS trial protocol: an international randomised controlled trial of intravenous sildenafil versus inhaled nitric oxide for the treatment of pulmonary hypertension in neonates with congenital diaphragmatic hernia
Source: BMJ Open. 2019 Nov 5;9(11):e032122. doi: 10.1136/bmjopen-2019-032122 (PMC6858099; doi:10.1136/bmjopen-2019-032122)
Supplement: Supplementary data [file bmjopen-2019-032122supp001.pdf]

Appendix:

CDH Euro Consortium:

Germany: Florian Kipfmueller, Department of Neonatology and Pediatric Critical Care Medicine, University Children's Hospital, Bonn. Spain: Maria Dolores Elorza, Ana Sanchez, Neonatology Department, Leopoldo Martinez, Pediatric Surgery Department, Carlos Labrandero, Viviana Arreo, Pediatric Cardiology Division, Hospital Universitario La Paz, Madrid. Africa Pertierra Cortada, Jordi Clotet Caba Neonatology Department, Hospital Sant Joan de Déu, Barcelona. Marta Aguar, Ana Gimeno, Raquel Escrig, Division of Neonatology, University and Polytechnic Hospital La Fe Valencia. Italy: Irma Capolupo, Pietro Bagolan, Department of Medical and Surgical Neonatology, Bambino Gesu' Children's Hospital, Rome. Fabrizio Ciralli, Genny Raffaeli, Giacomo Cavallaro, Valentina Condò, Fondazione IRCCS Ca' Granda Ospedale Maggiore Policlinico, NICU, University of Milan, Department of Clinical Sciences and Community Health. United Kingdom: United Kingdom - Paul D. Losty Department Of Paediatric Surgery, Division of Child Health, Alder Hey Children's Hospital NHS Foundation Trust, University of Liverpool , Marie Horan, Paediatric Intensive Care Alder Hey Children's Hospital NHS Foundation Trust, University of Liverpool. Nimish V. Subhedar, NICU, Liverpool Women's Hospital, Liverpool. Yogen Singh, Department of Neonatology, Cambridge University Hospitals NHS Foundation trust, Cambridge. Emma E. Williams, The Asthma UK Centre in Allergic Mechanisms of Asthma; Women and Children's Health, School of Life Course Sciences, Faculty of Life Sciences and Medicine, King's College London, Denmark Hill, London. Theodore Dassios, Ravindra Bhat, King's College Hospital NHS Foundation Trust, London. Austria: Jennifer B. Brandt, Alexandra Kreissl, Angelika Berger, Department of Pediatrics and Adolescent Medicine, Division of Neonatology, Pediatric Intensive Care Medicine and Neuropediatrics, Medical University of Vienna. Berndt Urlesberger, Division of Neonatology, Department of Pediatrics and Adolescent Medicine, Medical University of Graz. Sweden: Carmen Mesas Burgos, Björn Frenckner, Department of Pediatric Surgery, Björn Larrson, Pediatric Intensive Care Unit, Karolinska University Hospital, Stockholm. Portugal: Carla Pinto, Serviço de Cuidados Intensivos Pediátricos, Hospital Pediátrico, Centro Hospitalar e Universitário de Coimbra, Coimbra. Joana Saldaha, Department of Neonatology, Hospital de Santa Maria, Lisbon. Belgium: Anne Debeer, Anne Smits, Neonatology, University Hospitals Leuven, Leuven. Norway: Ragnhild Emblem, Department of Pediatric Surgery, Oslo University Hospital, Oslo. Canada: Richard Keijzer, Department of Surgery, Yassar Elsayed, Department of Neonatology, Pediatrics and Child Health, University of Manitoba and Children's Hospital Research Institute of Manitoba. Australia: David Tingay, Department of Neonatology, Royal Children's Hospital, Melbourne, Australia. The Netherlands: Ulrike Kraemer, Intensive Care and department of Pediatric Surgery, Erasmus MC, Rotterdam.
