# Supplementary material for: The CoDiNOS trial protocol: an international randomised controlled trial of intravenous sildenafil versus inhaled nitric oxide for the treatment of pulmonary hypertension in neonates with congenital diaphragmatic hernia
Source: BMJ Open. 2019 Nov 5;9(11):e032122. doi: 10.1136/bmjopen-2019-032122 (PMC6858099; doi:10.1136/bmjopen-2019-032122)
Supplement: Author's manuscript [file bmjopen-2019-032122.draft_revisions.pdf]

BMJ Open is committed to open peer review. As part of this commitment we make the peer review history of every article we publish publicly available.

When an article is published we post the peer reviewers' comments and the authors' responses online. We also post the versions of the paper that were used during peer review. These are the versions that the peer review comments apply to.

The versions of the paper that follow are the versions that were submitted during the peer review process. They are not the versions of record or the final published versions. They should not be cited or distributed as the published version of this manuscript.

BMJ Open is an open access journal and the full, final, typeset and author-corrected version of record of the manuscript is available on our site with no access controls, subscription charges or pay-per-view fees (http://bmjopen.bmj.com).

If you have any questions on BMJ Open's open peer review process please email info.bmjopen@bmj.com

BMJ Open

# The CoDiNOS trial protocol: An international randomized controlled trial of intravenous sildenafil versus inhaled nitric oxide for the treatment of pulmonary hypertension in neonates with congenital diaphragmatic hernia.

| | |
|---|---|
| Journal: | *BMJ Open* |
| Manuscript ID | bmjopen-2019-032122 |
| Article Type: | Protocol |
| Date Submitted by the Author: | 05-Jun-2019 |
| Complete List of Authors: | Cochius - den Otter, Suzan; Erasmus University Rotterdam, Pediatric Intensive Care<br>Schaible, Thomas; University Medical Center, Mannheim<br>Greenough, Anne; Kings College London, Department of Women and Children's Health, School of Life Sciences, Faculty of Life Science and Medicine<br>van Heijst, Arno; Radboudumc, Division of Neonatology, Department of Pediatrics<br>Patel, Neil; Royal Hospital for Children Glasgow, Departement of Neonatology<br>Allegaert, Karel; Erasmus MC Sophia, Pediatrics, Division of Neonatology<br>van Rosmalen, Joost; Erasmus MC, Biostatistics<br>Tibboel, Dick; Erasmus MC-Sophia Children's Hospital, Intensive Care and Pediatric Surgery |
| Keywords: | congenital diaphragmatic hernia, sildenafil, nitric oxide, THERAPEUTICS, pulmonary hypertension |
| | |

SCHOLARONE™
Manuscripts

**The CoDiNOS trial protocol: An international randomized controlled trial of intravenous sildenafil versus inhaled nitric oxide for the treatment of pulmonary hypertension in neonates with congenital diaphragmatic hernia.**

Suzan CM Cochius – den Otter MD[1], Thomas Schaible MD PhD[2], Anne Greenough MD[3], Arno van Heijst MD PhD[4], Neil Patel MD[5], Karel Allegaert MD PhD[1,6], Joost van Rosmalen PhD[7], Dick Tibboel MD PhD[1] on behalf of the CDH EURO Consortium

[1]Intensive Care and department of Pediatric Surgery, Erasmus MC, Rotterdam, the Netherlands. [2]Department of Neonatology Universitätsmedizin Mannheim, Mannheim, Germany. [3] The Asthma UK Centre in Allergic Mechanisms of Asthma; Women and Children's Health, School of Life Course Sciences, Faculty of Life Sciences and Medicine, King's College London, Denmark Hill, London, United Kingdom.[4] Division of Neonatology, Department of Pediatrics, Radboudumc Amalia Children's Hospital, Nijmegen, The Netherlands. [5]Departement of Neonatology, Royal Hospital for Children, Glasgow, United Kingdom. [6]Department of Development and Regeneration, KU, Leuven, Belgium. [7]Department of Biostatistics, Erasmus MC, Rotterdam, The Netherlands.

Corresponding author

Suzan CM Cochius – den Otter

Erasmus MC-Sophia Children's Hospital

Intensive Care Unit

Wytemaweg 80

3015 CN Rotterdam

Tel.: (+31) 10 7031769

Fax: (+31) 10 7037162

s.denotter@erasmusmc.nl

**Word count:** 3004

**Keywords:** Congenital Diaphragmatic Hernia, Pulmonary Hypertension, Therapeutics, Sildenafil, Nitric Oxide

For peer review only

## Abstract

### Introduction

Congenital diaphragmatic hernia (CDH) is a developmental defect of the diaphragm that impairs normal lung development, causing pulmonary hypertension (PH). PH in CDH newborns is the main determinant for morbidity and mortality. Different therapies are still mainly based on “trial and error”. Inhaled nitric oxide (iNO) is often the drug of first choice. However, iNO does not seem to improve mortality. Intravenous (iv) sildenafil has reduced mortality in newborns with PH without CDH, but prospective data in CDH patients are lacking.

### Methods and analysis

*Methods:* In an open label, multicenter, international randomized controlled trial 330 newborns with CDH and PH will be recruited over a four-year period (2018-2022). Patients are randomized for iv sildenafil or iNO. Sildenafil is given in a loading dose of 0.4 mg/kg in 3 hours; followed by continuous infusion of 1.6 mg/kg/day, iNO is dosed at 20 ppm.

Primary outcome is absence of PH on day 14 without pulmonary vasodilator therapy and/or absence of death within the first 28 days of life. Secondary outcome measures include clinical and echocardiographic markers of PH in the first year of life.

*Data analysis:* We hypothesize that sildenafil gives a 25% reduction in PH from 68% to 48% on day 14, for which a sample size of 330 patients is needed. An intention-to-treat analysis will be performed. A p-value (two-sided) < 0.05 is considered significant in all analyses.

### Ethics and dissemination

Ethics approval has been granted by the ethics committee in Rotterdam (MEC-2017-324) and the central Committee on Research Involving Human Subjects (NL60229.078.17) in the Netherlands. The principles of the Declaration of Helsinki, the Medical Research Involving Human Subjects Act, and the national rules and regulations on personal data protection will be used. Parental informed consent will be obtained.

### Registration

Trial registration number NTR6982 (Trial NL6796).


For peer review only



For peer review only - http://bmjopen.bmj.com/site/about/guidelines.xhtml

## Article summary

### Strengths and limitations of this study

- **The CoDiNOS trial is the first randomized controlled multicenter trial to evaluate the effect of intravenous sildenafil and compare with iNO on pulmonary hypertension in newborns with CDH.**

- **This is the second randomized controlled trial of the CDH EURO Consortium in a large group of newborns with this orphan disease.**

- **Treatment allocation is not blinded in the trial. This is not feasible because of variability in iNO equipment and gas mixtures use. Instead, the researchers who analyze the echocardiography to evaluate PH will be blinded to the treatment.**

- **There is no non-intervention group, as it is common practice in the centers of the CDH EURO Consortium to give iNO; hence, it is considered unethical to withhold treatment for one group.**

For peer review only



For peer review only - http://bmjopen.bmj.com/site/about/guidelines.xhtml

**Introduction:**

Congenital diaphragmatic hernia (CDH) is a severe developmental defect of the diaphragm with a mortality of 27% in live-born patients [1]. Because of this defect, the abdominal organs herniate into the chest causing pulmonary hypoplasia and abnormal pulmonary vasculature growth, resulting in pulmonary hypertension (PH) [2]. In adults and children, PH is defined as mean pulmonary artery pressure (mPAP) exceeding 25 mmHg with a pulmonary capillary wedge pressure of minimal 15 mmHg [3].

The normal pulmonary vascular transition of the neonate takes around two months to achieve these low values of mPAP. During fetal life, there is high resistance in the pulmonary circulation which results in most of the blood flow to bypass the lungs through the ductus arteriosus and oval foramen. Immediately after birth, the pulmonary vascular resistance drops and the blood flow to the lungs significantly increases [4]. In contrast, the pulmonary vascular resistance often does not drop adequately in children with CDH due to a decreased vascular bed associated with lung hypoplasia, and an altered development of the pulmonary vasculature with excessive muscularization of the arterioles, with increased thickness of the arterial media and adventitia. The incidence of PH in CDH patients is 68-79% and causes considerable morbidity and mortality [1, 2, 5]. Therapy in newborns with PH , such as inhaled nitric oxide (iNO) and sildenafil, has improved outcomes in general. However, trials in infants with CDH are sparse.

Inhaled nitric oxide (iNO) diffuses rapidly across the alveolar-capillary membrane into the smooth muscle of pulmonary vessels to activate soluble guanylate cyclase. This enzyme mediates many of the biological effects of iNO, and is responsible for the conversion of GTP to cGMP. The increase of intracellular cGMP relaxes smooth muscles via several mechanisms. iNO also causes bronchodilation and has anti-inflammatory and anti-proliferative effects [6]. In term and near term infants with persistent pulmonary hypertension of the newborn (PPHN), iNO decreases the median duration of mechanical ventilation and reduces the need for extracorporeal membrane oxygenation (ECMO). However, in the two available randomized controlled trials (RCT) with a small number of patients with CDH, mortality did not improve and more ECMO treatment was needed [7, 8]. In the centers of the CDH EURO Consortium, iNO is standard of care in infants with CDH and PH although the positive pharmacodynamic effects in these infants are less convincing then in infants with PPHN [5, 9]. The pathophysiological mechanism of this difference is not understood.

Sildenafil is a selective phosphodiesterase type 5 (PDE5) inhibitor. PDE5 is an enzyme that specifically degrades cGMP. Sildenafil inhibits PDE5, increasing cGMP and NO-mediated vasodilatation of the smooth muscles in arteries. Only five RCTs have been performed in newborns, all non-CDH patients



For peer review only - http://bmjopen.bmj.com/site/about/guidelines.xhtml

with PPHN. Four of these studies showed a decrease in oxygenation index (OI) and mortality in a setting where iNO was not available, while one trial showed no additional benefit of sildenafil when added to iNO [10]. Although sildenafil is increasingly used in CDH patients, only retrospective data are available [11]. A decrease in pulmonary vascular resistance index and an increase in cardiac output were found in a small group of oral sildenafil-treated infants with CDH refractory to iNO [12]. Intravenous sildenafil improved OI and reversed the right-to-left shunt ratio over the PDA, but it also increased the need for inotropic support [13, 14]. However, its effect on outcome is unknown.

We hypothesize that intravenous sildenafil is superior to iNO and should be the first line of treatment for PH in CDH patients. iNO is the therapy of first choice in most centers despite the lack of evidence, and sildenafil is the most promising drug for the treatment of PH in CDH patients and is increasingly being used [5, 11, 15]. However, no studies have been performed comparing iNO with intravenous sildenafil in newborns with CDH and PH or PH alone. Based on the current knowledge, there is equipoise for both treatment modalities.

For peer review only



For peer review only - http://bmjopen.bmj.com/site/about/guidelines.xhtml

**Methods and analysis:**

**Design**

The CoDiNOS trial is a prospective, multicenter, international randomized controlled trial conducted in high volume pediatric surgical centers in Europe, Canada and Australia. The members of the CDH Euro Consortium participating in the trial are listed in the Appendix.

**Objectives**

The primary objective of the study is to determine whether the incidence of PH is lower in CDH patients treated with intravenous sildenafil than in patients treated with iNO, defined as the absence of PH on echocardiography on day 14 without pulmonary vasodilator therapy and without treatment failure and/or death within the first 28 days after birth. PH is defined as systolic pulmonary arterial pressure> 2/3 systolic systemic pressure and/or right ventricular (RV) dilatation/septal displacement and RV dysfunction +/- left ventricular dysfunction.

The secondary outcomes are:

(1) change in OI after 12 and 24 hours of therapy

(2) overall mortality

(3) the incidence of treatment failure which is defined as:

- inability to maintain preductal saturations above 85% (± 7 kPa or 52 mmHg) or postductal saturations above 70% (±5.3 kPa or 40 mmHg)
- and/or increase in $CO_2$ > 70 mmHg (9.3 kPa) despite optimization of ventilator management
- and/or inadequate oxygen delivery with metabolic acidosis defined as lactate ≥ 5 mmol/l and pH < 7.15 and/or hypotension resistant to fluid therapy and adequate inotropic support resulting in a urine output < 0.5 ml/kg/hour
- and/or lactate ≥ 5 mmol/l and pH < 7.15
- and/or OI consistently ≥ 40

(4) time on intervention drug, defined as intervention drug free days after initiation of the intervention, calculated on day 14

(5) need for ECMO



For peer review only - http://bmjopen.bmj.com/site/about/guidelines.xhtml

(6) ventilator free days on day 28

(7) the use of drugs for PH treatment during the hospital admission

(8) the use of pulmonary and/or cardiac medication at discharge and its total duration of administration

(9) short-term and long-term PH on echocardiography at 24 hours, 28 days/discharge and 6 and 12 months

(10) the incidence of chronic lung disease

(11) the development of neurological abnormalities evaluated with ultrasound of the brain before the start of the trial, after surgery and before discharge

(12) the external validation of the sildenafil PKPD model for the pharmacokinetics and the pharmacodynamic effects of sildenafil

Safety outcomes include adverse events due to the study drugs and the vasoactive-inotropic support score (VIS).

**Patients**

Infants diagnosed with CDH who have PH in the first week after birth, are eligible for the trial if born at or after a gestational age of 34 weeks. The diagnosis of PH is defined as at least two of the following four criteria: (I) systolic pulmonary arterial pressure> 2/3 systolic systemic pressure estimated by echocardiography. (II) RV dilatation/septal displacement, RV dysfunction +/- left ventricular dysfunction. (III) Pre-post ductal $SpO_2$ difference > 10%. (IV) OI >20. Exclusion criteria are a severe chromosomal anomaly which may imply a decision to stop or not to start life-saving medical treatment, severe cardiac anomaly expected to need corrective surgery in the first 60 days of life, renal anomalies associated with oligohydramnios, severe orthopedic and skeletal deformities, which are likely to influence thoracic, and / or lung development and severe anomalies of the central nervous system. Patients who are born in another center and transported with iNO are also excluded from the trial. Patients who received fetal interventions (trachea balloon placement) are not excluded.

Following antenatal diagnosis, the parents are counselled and informed about the study. Also, they receive a patient information letter and an informed consent form . If the patient is not born in a participating center or the diagnosis of CDH was not known, parents are counselled after the diagnosis of CDH and are informed about the study. Also, they receive written information and an



For peer review only - http://bmjopen.bmj.com/site/about/guidelines.xhtml

informed consent form. This informed consent form contains consent for the trial and for collection of data and material for future research.

For the development of the protocol the SPIRIT reporting guidelines have been used [16]. This publication is based on protocol version 4, June 13th 2018.

**Patient and public involvement**

Patients and the public were not involved in the development of the trial protocol. However, CDH UK Sparks, as a parent organization, has assessed and commented on the protocol and as provided start-up funding as also mentioned in the funding statement. This organization is and will be regularly informed on progress and results of the trial.

**Study procedures**

*Baseline assessment*

Antenatal ultrasound data about the characteristics of the CDH are collected. These data include the observed/expected lung-head ratio, position of the liver and stomach and the amniotic fluid index. An MRI or an ultrasound is performed depending on local experience and possibilities. If an MRI is performed, the observed/expected fetal lung volume will be calculated. Also data on prenatal interventions are collected. In all mothers, a planned vaginal or caesarean delivery is pursued.

*Randomization, intervention and blinding*

When meeting the inclusion criteria, the patients are randomized with computer generated concealed allocation, made by the independent statistician of the Data Safety and Monitoring Board. Blocked randomization with stratification by center is used to achieve equal distribution of the two interventions among the participants.

Postnatally, infants are treated according to a standardized protocol for patients with CDH, which is implemented in all participating centers. This protocol was developed with the available evidence and consensus between the participating centers and was updated in June 2016 [16, 22]. If the patient is diagnosed with PH in the first week of life, the patient will be allocated to one of the two study drugs (figure 1). iNO is provided by a tank connected to a ventilator. Different devices are used in different centers. Some centers use integrated systems, making it impossible to disconnect the iNO tank and replace it with another gas to facilitate a blinded intervention. Therefore, the study is open label. iNO is given with a starting dose of 20 ppm, which is the maximum dose [17, 18]. Sildenafil is given intravenously, using a loading dose of 0.4mg/kg in 3 hours, followed by continuous infusion of 1.6mg/kg/day [19, 20]. To wean the study drugs a standard protocol is followed (figure 2).



For peer review only - http://bmjopen.bmj.com/site/about/guidelines.xhtml

The allocated drugs will be restarted as per protocol if criteria for its use are met again before the age of 14 days. After day 14 treatment of PH will be at the discretion of the local medical team and the study drug can be changed to, for instance, sildenafil orally. The use of bosentan next to the study treatment is allowed.

The intervention will be prematurely stopped when the patient meets one or more of the defined failure criteria, described at point three of the secondary outcomes. Further treatment will then be at the discretion of the medical team and iNO and sildenafil can both be given outside the study protocol. An ECMO-procedure may then be started in centers where ECMO is available. Data of all patients are used in the intention-to-treat analysis.

*Follow up*

After day 14, additional clinical data, such as time on ventilator support (days) and the use of drugs for the treatment of PH, are collected to answer the secondary outcome questions.  Also, echocardiographic measurements are taken at 6 and 12 months to evaluate the presence of chronic PH (table 1)

Table 1 Procedures and measurements

| | Day 0-7 before start thera-py | 3 hrs after start silde-nafil | 12hrs after start | 8 am after start | 24hrs after start | Day of surgery, pre-opera-tively | Day after sur-gery | Day of ECMO, pre-cannula-tion | 8 am after start ECMO | Day 14 | Day 28 / before dis-charge | Day 56 | 6 mnth | 12 mnth |
|---|---|---|---|---|---|---|---|---|---|---|---|---|---|---|
| Echocardio-graphy | X | | | | X | | | | | X | X | | X | X |
| Calculation OI | X | | X | | X | | | | | | | | | |
| Calculation VIS score | X | | X | | X | | | | | | | | | |
| Blood sample | X | | | X | | X | X | X | X | X | | | | |
| Tracheal aspirate | X | | | X | | X | X | X | X | X | | | | |
| Urine sample | X | | | X | | X | X | X | X | X | | | | |
| Severity of CLD | | | | | | | | | | | X | X | | |
| Ultrasound brain | X | | | | | | X | | | | | | | |
| Sildenafil plasma level | | X | | X | | X | X | | X | | | | | |

OI: oxygenation index; VIS score: vasoactive-inotropic support score; CLD: chronic lung disease; ECMO: extracorporeal membrane oxygenation

For peer review only



For peer review only - http://bmjopen.bmj.com/site/about/guidelines.xhtml

*Data collection*

Echocardiography parameters are measured by local physicians, centrally collected and reviewed by two blinded independent physicians to reduce inter-observer variation. Demographic and neonatal characteristics as well as data on the clinical course of all patients are entered in a password protected web-based database in Rotterdam (OpenClinica). Upon request the collected data will be available. All centers will keep a logbook of the number of non-participants, including the reasons for not participating. Study documents are securely stored at each study site for 15 years.

*Laboratory testing*

Blood, urine and tracheal samples are collected in most centers during the trial. Blood samples are collected before the start of the study and at different time points until day 14. Some samples will be used to externally validate a NONMEM prediction model for sildenafil. The other samples will be used in future research on biomarkers to predict severity and outcome of PH in CDH patients. The samples are centrifuged for 6 minutes at 3000 rpm [21]. Thereafter, the plasma is removed and stored at –20 degrees Celsius or colder. The total amount of blood taken is maximal 2.5 % of the circulating volume. Blood sampling will only be done if a central or peripheral line is still present and/or in combination with routine laboratory measurements. This way blood sampling is a minimal burden for the patient.

Tracheal aspirate for proteomic analysis is also collected at different time points during routine tracheal suctioning in ventilated patients. Protein profiling with proteomics is used to identify specific groups of proteins that are involved in the pathogenesis of PH. The tracheal aspirates is centrifuged for 6 minutes at 3000 rpm and stored at –80 degrees Celsius [43].

Also, 8-hour urine is collected at different time points. Two samples of 5 ml are taken and stored at –20 degrees Celsius or colder.

*Withdrawal of participants*

Parents may decide to withdraw from the study at any time without any consequences. The investigator can decide to withdraw a patient from the study for urgent medical reasons. In some cases, there may be exclusion criteria, which were not known before randomization. If this is the case, the patient will be withdrawn from the study after contacting the study coordinator. With consent of the parents data will still be collected, stored and analyzed to perform an intention-to-treat analysis. These children will be treated according to standard practice [9, 15].



For peer review only - http://bmjopen.bmj.com/site/about/guidelines.xhtml

**Sample size calculation**

We powered our study using PH at day 14 as primary outcome. Lusk et al. showed that PH in CDH patients on day 14 has a positive predictive value of 0.8 for death, death or ventilation, and death or ventilator support. PH on day 14 is observed in 64% of CDH patients [22].

If we assume a 25% relative reduction of PH to 48%, a sample size of 300 patients (150 patients per group) is needed to obtain a power of 80%. This will match a number needed to treat of 6.25. Taking missing data and the effects of correction for covariates into account, we adjust this sample size to 330 patients. In the collaborating centers 550 patients will be born in three years. Based on our earlier trial (VICI trial) we expect to have an inclusion rate of 60%. Therefore, the inclusion of 330 patients should be reached in three years.

**Data analysis**

The patients will be analyzed according to the group they are randomized to (intention-to-treat analysis). A p-value (two-sided) < 0.05 is considered significant in all analyses. The primary endpoint PH will be analyzed using multiple logistic regression with randomization arm, center, observed/expected head-lung ratio, position of the liver, side of the defect, defect size and ventilation modality as independent variables [23]. If necessary, multiple imputation using the fully conditional specification method will be used to account for missing data in the independent variables.

The following analyses will be performed for the secondary outcomes. The distribution of VIS score in all study participants will be compared between t=0 and t=12 hours after initiation of drug administration using a Wilcoxon signed rank test. The distribution of changes in OI and VIS score from t=0 to t=12 and t=24 hours will be compared between the randomization groups with a Mann-Whitney test. The overall mortality in the first year of life will be compared between the randomization groups with Kaplan-Meier curves and the log-rank test. The number of treatment failures, the need for ECMO (in ECMO centers), and the need for medication for pulmonary hypertension or chronic lung disease at discharge, and during the first year of life, will be compared between randomization groups with chi-square tests. The number of study drug free days at day 14, the number of ventilation-free days until day 28, the fraction of days with need for medical treatment (excluding the study drug) for PH during the hospital admission, and the severity of chronic lung disease using the Bancalari definition, will be compared between randomization groups with Mann-Whitney tests. Deaths will be counted as the worst outcome in these analyses, in accordance with the intention-to-treat principle. The presence of pulmonary hypertension at 28



For peer review only - http://bmjopen.bmj.com/site/about/guidelines.xhtml

days/discharge, 6 and 12 months according to the echocardiographic parameters will be compared between randomization groups with a chi-square test.

To externally validate the pharmacokinetic model of sildenafil and it active metabolite (built in NONMEM) Normalized prediction distribution errors (NPDE) and Visual Predictive Check (VPC) will be used. Furthermore, the model will be used to predict the drug concentrations from the new data set  using simulations, in which we expect that the difference will be less than 20%. To find a relationship between the concentration of sildenafil, its active metabolite and the clinical effects, such as OI, VIS score and echocardiography measures, a Mann-Whitney or T test will be used.

**Safety reporting and trial oversight**

All severe adverse events (SAEs) and suspected unexpected serious adverse reactions (SUSARs) are reported from the enrolment until 12 month follow-up. Persistent or significant disability or incapacity that was not expected with the given O/E LHR is evaluated as an SAE. An elective hospital admission is not a SAE.  All SAEs and SUSARs are reported to the approving ethics committees in accordance with their requirements. We will report the SAEs and SUSARs that result in death or are life threatening within 7 days of first knowledge. All other SAEs and SUSARs will be reported within a period of maximum 15 days. Once a year throughout the clinical trial, we will submit a safety report to the approving ethics committees and competent authorities of the countries involved.

The data safety monitoring board will monitor the incidence of mortality on a continuous basis. If at some point a large difference in mortality between the two treatment groups is noticed, the data safety monitoring board may recommend ending the study.

Insurance will cover compensation to patients who suffer harm from trial participation.

**Ethics and dissemination**

Ethics approval has been granted by the local ethics committee in Rotterdam (MEC-2017-324) and by the central Committee on Research Involving Human Subjects (NL60229.078.17) in the Netherlands. Important amendments will be communicated to all relevant parties.  The study will be conducted according to the principles of the Declaration of Helsinki, in accordance with the Medical Research Involving Human Subjects Act, and national rules and regulations on personal data protection. Parental informed consent will be obtained. The results of this study will be disseminated via peer-reviewed publications and implemented in the international guidelines for the treatment of newborns with CDH.

For peer review only



For peer review only - http://bmjopen.bmj.com/site/about/guidelines.xhtml

## Literature


1. Snoek, K.G., et al., *Conventional Mechanical Ventilation Versus High-frequency Oscillatory Ventilation for Congenital Diaphragmatic Hernia: A Randomized Clinical Trial (The VICI-trial).* Ann Surg, 2015.
2. Robinson, P.D. and D.A. Fitzgerald, *Congenital diaphragmatic hernia.* Paediatr Respir Rev, 2007. **8**(4): p. 323-34; quiz 334-5.
3. Galie, N., et al., *Guidelines for the diagnosis and treatment of pulmonary hypertension: the Task Force for the Diagnosis and Treatment of Pulmonary Hypertension of the European Society of Cardiology (ESC) and the European Respiratory Society (ERS), endorsed by the International Society of Heart and Lung Transplantation (ISHLT).* Eur Heart J, 2009. **30**(20): p. 2493-537.
4. Vali, P. and S. Lakshminrusimha, *The Fetus Can Teach Us: Oxygen and the Pulmonary Vasculature.* Children (Basel), 2017. **4**(8).
5. Putnam, L.R., et al., *Evaluation of Variability in Inhaled Nitric Oxide Use and Pulmonary Hypertension in Patients With Congenital Diaphragmatic Hernia.* JAMA Pediatr, 2016.
6. Ichinose, F., J.D. Roberts, Jr., and W.M. Zapol, *Inhaled nitric oxide: a selective pulmonary vasodilator: current uses and therapeutic potential.* Circulation, 2004. **109**(25): p. 3106-11.
7. *Inhaled nitric oxide and hypoxic respiratory failure in infants with congenital diaphragmatic hernia. The Neonatal Inhaled Nitric Oxide Study Group (NINOS).* Pediatrics, 1997. **99**(6): p. 838-45.
8. Clark, R.H., et al., *Low-dose nitric oxide therapy for persistent pulmonary hypertension of the newborn. Clinical Inhaled Nitric Oxide Research Group.* N Engl J Med, 2000. **342**(7): p. 469-74.
9. Reiss, I., et al., *Standardized postnatal management of infants with congenital diaphragmatic hernia in Europe: the CDH EURO Consortium consensus.* Neonatology, 2010. **98**(4): p. 354-64.
10. Kelly, L.E., A. Ohlsson, and P.S. Shah, *Sildenafil for pulmonary hypertension in neonates.* Cochrane Database Syst Rev, 2017. **8**: p. CD005494.
11. Lakshminrusimha, S., et al., *Milrinone in congenital diaphragmatic hernia - a randomized pilot trial: study protocol, review of literature and survey of current practices.* Matern Health Neonatol Perinatol, 2017. **3**: p. 27.
12. Noori, S., et al., *Cardiovascular effects of sildenafil in neonates and infants with congenital diaphragmatic hernia and pulmonary hypertension.* Neonatology, 2007. **91**(2): p. 92-100.
13. Bialkowski, A., F. Moenkemeyer, and N. Patel, *Intravenous sildenafil in the management of pulmonary hypertension associated with congenital diaphragmatic hernia.* Eur J Pediatr Surg, 2015. **25**(2): p. 171-6.
14. Kipfmueller, F., et al., *Continuous intravenous sildenafil as an early treatment in neonates with congenital diaphragmatic hernia.* Pediatr Pulmonol, 2018.
15. Snoek, K.G., et al., *Standardized Postnatal Management of Infants with Congenital Diaphragmatic Hernia in Europe: The CDH EURO Consortium Consensus - 2015 Update.* Neonatology, 2016. **110**(1): p. 66-74.
16. Chan, A.W., et al., *SPIRIT 2013 statement: defining standard protocol items for clinical trials.* Ann Intern Med, 2013. **158**(3): p. 200-7.
17. Tworetzky, W., et al., *Inhaled nitric oxide in neonates with persistent pulmonary hypertension.* Lancet, 2001. **357**(9250): p. 118-20.
18. Barrington, K.J., et al., *Nitric oxide for respiratory failure in infants born at or near term.* Cochrane Database Syst Rev, 2017. **1**: p. CD000399.
19. Steinhorn, R.H., et al., *Intravenous sildenafil in the treatment of neonates with persistent pulmonary hypertension.* J Pediatr, 2009. **155**(6): p. 841-847 e1.




For peer review only - http://bmjopen.bmj.com/site/about/guidelines.xhtml

20. Mukherjee, A., et al., *Population pharmacokinetics of sildenafil in term neonates: evidence of rapid maturation of metabolic clearance in the early postnatal period.* Clin Pharmacol Ther, 2009. **85**(1): p. 56-63.
21. Snoek, K.G., et al., *High-Sensitivity Troponin T and N-Terminal Pro-Brain Natriuretic Peptide in Prediction of Outcome in Congenital Diaphragmatic Hernia: Results from a Multicenter, Randomized Controlled Trial.* J Pediatr, 2016. **173**: p. 245-249 e4.
22. Lusk, L.A., et al., *Persistence of pulmonary hypertension by echocardiography predicts short-term outcomes in congenital diaphragmatic hernia.* J Pediatr, 2015. **166**(2): p. 251-6 e1.
23. Lally, K.P., et al., *Standardized reporting for congenital diaphragmatic hernia--an international consensus.* J Pediatr Surg, 2013. **48**(12): p. 2408-15.

**Author contributions**

All investigators of the Consortium described below, have contributed to the design of the trial protocol and have approved this version for submission. Coordinating investigator S Cochius – den Otter and Prof D Tibboel are responsible for all aspects of the study conduct, practically study oversight, recruitment, training of the participating hospitals, reporting of the SAEs and SUSARs, outcome assessment and data management. Prof D Tibboel, Prof K Allegaert, Dr T Schaible, Dr A van Heijst, Dr A Greenough and Dr N Patel are responsible for study oversight. J van Rosmalen has contributed to statistical methods and will be involved in interpretation of the results. S Cochius- den Otter will lead the dissemination and translation of results with the contribution of all investigators of the CDH EURO Consortium. Also all members will have authority over the data.

**Funding statement**

This work was supported by CDH UK Sparks grant number 16EMC01 and by Stichting Sophia Kinderziekenhuis Fonds grant number S17-19.

**Competing interests**

None

**Figure 1** Trial flow chart

Flow chart showing the steps of the trial, from birth until 12 months. CDH: congenital diaphragmatic hernia; SAE: serious adverse event; SUSAR: suspected unexpected serious adverse event

**Figure 2** Protocol to wean study drug

Flow chart showing the protocol to wean off inhaled nitric oxide or intravenous sildenafil. iNO: inhaled nitric oxide; ppm: parts per million

For peer review only

Figure 1 Trial flow chart

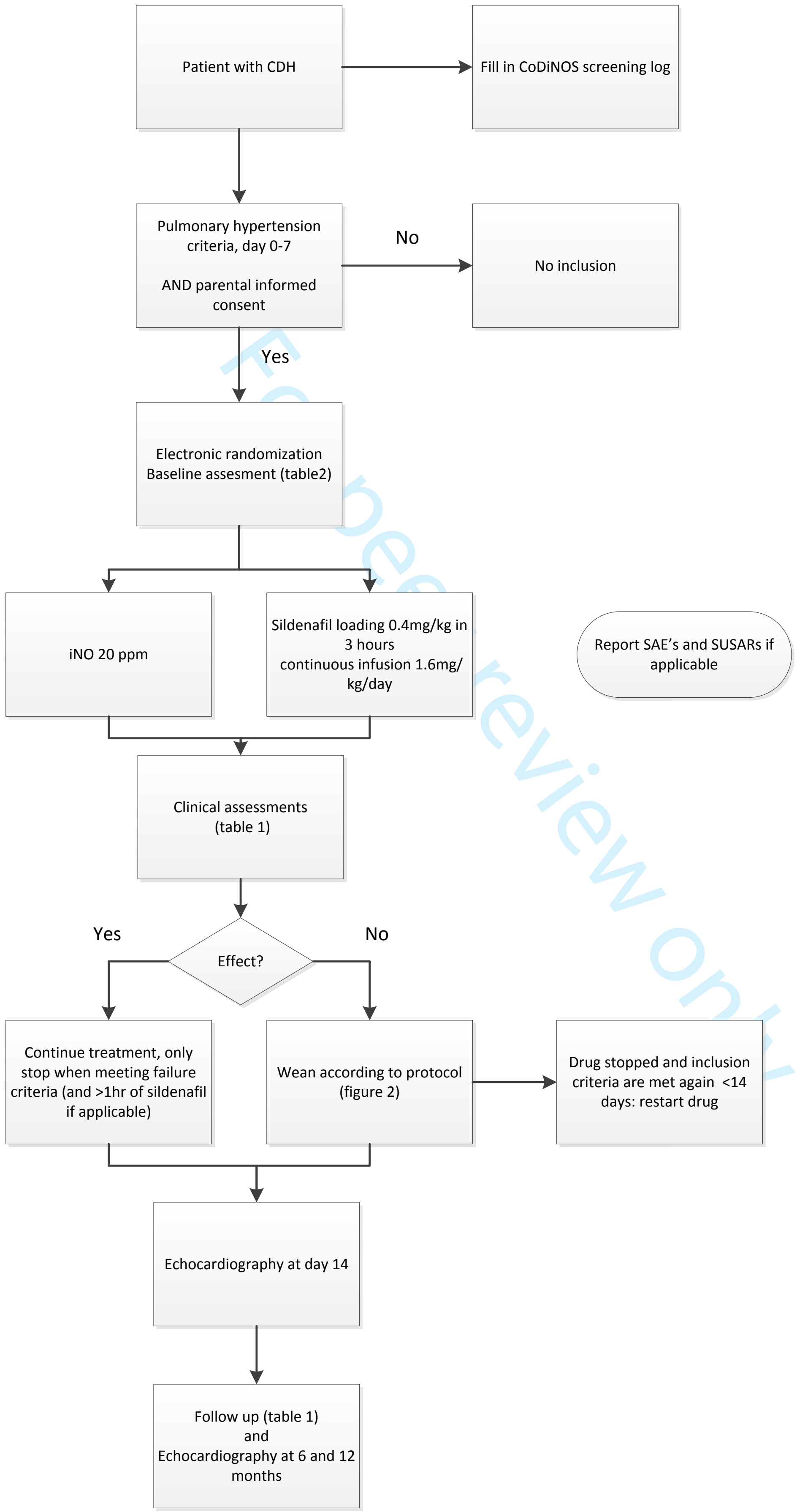


CDH: congenital diaphragmatic hernia; SAE: serious adverse event; SUSAR: suspected unexpected serious adverse event

For peer review only - http://bmjopen.bmj.com/site/about/guidelines.xhtml

Figure 2 Protocol to wean study drug

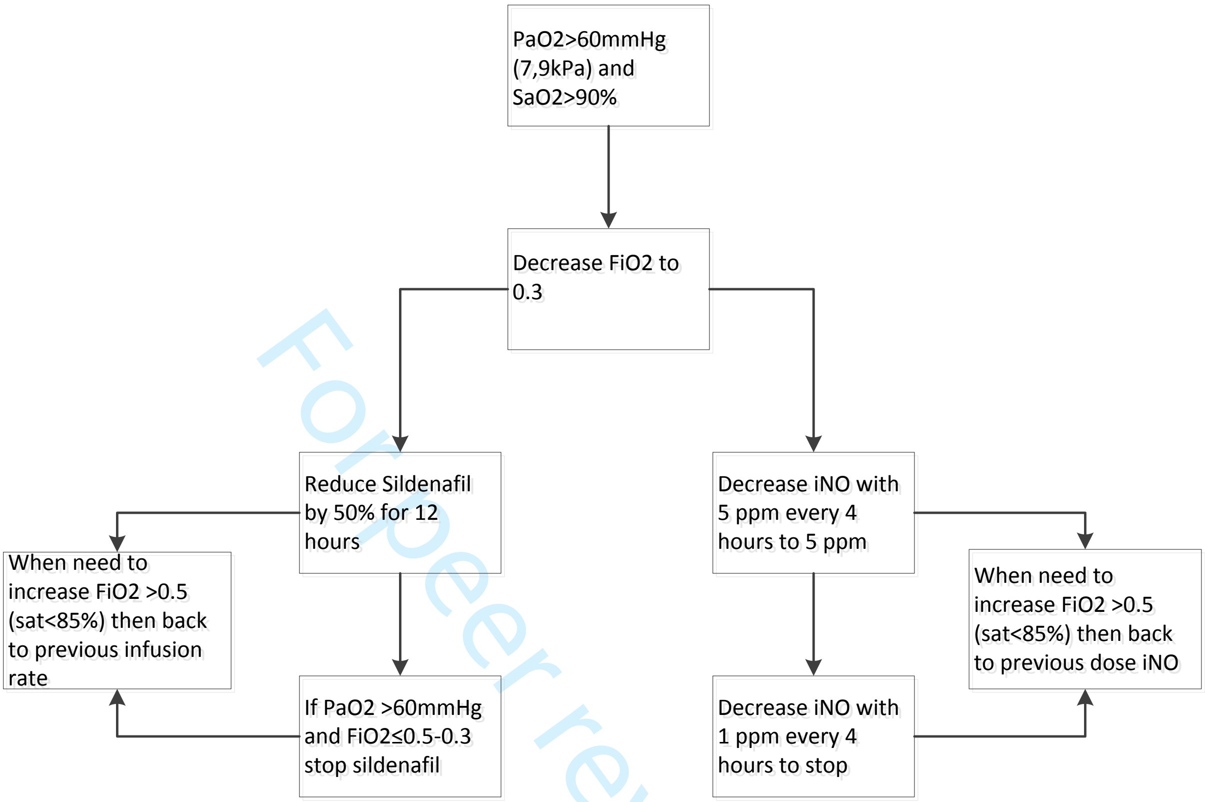


For peer review only

**Appendix:**

CDH Euro Consortium:

Germany: Florian Kipfmueller, Department of Neonatology and Pediatric Critical Care Medicine, University Children's Hospital, Bonn. Spain: Maria Dolores Elorza, Ana Sanchez, Neonatology Department, Leopoldo Martinez, Pediatric Surgery Department, Carlos Labrandero, Viviana Arreo, Pediatric Cardiology Division, Hospital Universitario La Paz, Madrid. Africa Pertierra Cortada, Jordi Clotet Caba Neonatology Department, Hospital Sant Joan de Déu, Barcelona. Marta Aguar, Ana Gimeno, Raquel Escrig, Division of Neonatology, University and Polytechnic Hospital La Fe Valencia. Italy: Irma Capolupo, Pietro Bagolan, Department of Medical and Surgical Neonatology, Bambino Gesu' Children's Hospital, Rome. Fabrizio Ciralli, Genny Raffaeli, Giacomo Cavallaro, Valentina Condò, Fondazione IRCCS Ca' Granda Ospedale Maggiore Policlinico, NICU, University of Milan, Department of Clinical Sciences and Community Health. United Kingdom: United Kingdom - Paul D. Losty Department Of Paediatric Surgery, Division of Child Health, Alder Hey Children's Hospital NHS Foundation Trust, University of Liverpool , Marie Horan, Paediatric Intensive Care Alder Hey Children's Hospital NHS Foundation Trust, University of Liverpool. Nimish V. Subhedar, NICU, Liverpool Women's Hospital, Liverpool. Yogen Singh, Department of Neonatology, Cambridge University Hospitals NHS Foundation trust, Cambridge. Emma E. Williams, The Asthma UK Centre in Allergic Mechanisms of Asthma; Women and Children's Health, School of Life Course Sciences, Faculty of Life Sciences and Medicine, King's College London, Denmark Hill, London. Theodore Dassios, Ravindra Bhat, King's College Hospital NHS Foundation Trust, London. Austria: Jennifer B. Brandt, Alexandra Kreissl, Angelika Berger, Department of Pediatrics and Adolescent Medicine, Division of Neonatology, Pediatric Intensive Care Medicine and Neuropediatrics, Medical University of Vienna. Berndt Urlesberger, Division of Neonatology, Department of Pediatrics and Adolescent Medicine, Medical University of Graz. Sweden: Carmen Mesas Burgos, Björn Frenckner, Department of Pediatric Surgery, Björn Larrson, Pediatric Intensive Care Unit, Karolinska University Hospital, Stockholm. Portugal: Carla Pinto, Serviço de Cuidados Intensivos Pediátricos, Hospital Pediátrico, Centro Hospitalar e Universitário de Coimbra, Coimbra. Joana Saldaha, Department of Neonatology, Hospital de Santa Maria, Lisbon. Belgium: Anne Debeer, Anne Smits, Neonatology, University Hospitals Leuven, Leuven. Norway: Ragnhild Emblem, Department of Pediatric Surgery, Oslo University Hospital, Oslo. Canada: Richard Keijzer, Department of Surgery, Yassar Elsayed, Department of Neonatology, Pediatrics and Child Health, University of Manitoba and Children's Hospital Research Institute of Manitoba. Australia: David Tingay, Department of Neonatology, Royal Children's Hospital, Melbourne, Australia. The Netherlands: Ulrike Kraemer, Intensive Care and department of Pediatric Surgery, Erasmus MC, Rotterdam.

For peer review only

# Reporting checklist for protocol of a clinical trial.

Based on the SPIRIT guidelines.

## Instructions to authors

Complete this checklist by entering the page numbers from your manuscript where readers will find each of the items listed below.

Your article may not currently address all the items on the checklist. Please modify your text to include the missing information. If you are certain that an item does not apply, please write "n/a" and provide a short explanation.

Upload your completed checklist as an extra file when you submit to a journal.

In your methods section, say that you used the SPIRIT reporting guidelines, and cite them as:

Chan A-W, Tetzlaff JM, Altman DG, Laupacis A, Gøtzsche PC, Krleža-Jerić K, Hróbjartsson A, Mann H, Dickersin K, Berlin J, Doré C, Parulekar W, Summerskill W, Groves T, Schulz K, Sox H, Rockhold FW, Rennie D, Moher D. SPIRIT 2013 Statement: Defining standard protocol items for clinical trials. Ann Intern Med. 2013;158(3):200-207

| | | Reporting Item | Page Number |
|---|---|---|---|
| Title | #1 | Descriptive title identifying the study design, population, interventions, and, if applicable, trial acronym | 1 |
| Trial registration | #2a | Trial identifier and registry name. If not yet registered, name of intended registry | 2 |
| Trial registration: data set | #2b | All items from the World Health Organization Trial Registration Data Set | 8 |
| Protocol version | #3 | Date and version identifier | 8 |
| Funding | #4 | Sources and types of financial, material, and other support | 14 |
| Roles and responsibilities: contributorship | #5a | Names, affiliations, and roles of protocol contributors | 14 and appendix |
| Roles and | #5b | Name and contact information for the trial sponsor | 1 |

For peer review only

| | | | |
|---|---|---|---|
| responsibilities: sponsor contact information | | | |
| Roles and responsibilities: sponsor and funder | #5c | Role of study sponsor and funders, if any, in study design; collection, management, analysis, and interpretation of data; writing of the report; and the decision to submit the report for publication, including whether they will have ultimate authority over any of these activities | 14 |
| Roles and responsibilities: committees | #5d | Composition, roles, and responsibilities of the coordinating centre, steering committee, endpoint adjudication committee, data management team, and other individuals or groups overseeing the trial, if applicable (see Item 21a for data monitoring committee) | 14 |
| Background and rationale | #6a | Description of research question and justification for undertaking the trial, including summary of relevant studies (published and unpublished) examining benefits and harms for each intervention | 4-5 |
| Background and rationale: choice of comparators | #6b | Explanation for choice of comparators | 4-5 |
| Objectives | #7 | Specific objectives or hypotheses | 6 |
| Trial design | #8 | Description of trial design including type of trial (eg, parallel group, crossover, factorial, single group), allocation ratio, and framework (eg, superiority, equivalence, non-inferiority, exploratory) | 6 |
| Study setting | #9 | Description of study settings (eg, community clinic, academic hospital) and list of countries where data will be collected. Reference to where list of study sites can be obtained | 6, 14, appendix |
| Eligibility criteria | #10 | Inclusion and exclusion criteria for participants. If applicable, eligibility criteria for study centres and | 7 |

For peer review only

| | | | |
|---|---|---|---|
| | | individuals who will perform the interventions (eg, surgeons, psychotherapists) | |
| Interventions: description | #11a | Interventions for each group with sufficient detail to allow replication, including how and when they will be administered | 8 |
| Interventions: modifications | #11b | Criteria for discontinuing or modifying allocated interventions for a given trial participant (eg, drug dose change in response to harms, participant request, or improving / worsening disease) | 9 |
| Interventions: adherance | #11c | Strategies to improve adherence to intervention protocols, and any procedures for monitoring adherence (eg, drug tablet return; laboratory tests) | n/a |
| Interventions: concomitant care | #11d | Relevant concomitant care and interventions that are permitted or prohibited during the trial | 8 |
| Outcomes | #12 | Primary, secondary, and other outcomes, including the specific measurement variable (eg, systolic blood pressure), analysis metric (eg, change from baseline, final value, time to event), method of aggregation (eg, median, proportion), and time point for each outcome. Explanation of the clinical relevance of chosen efficacy and harm outcomes is strongly recommended | 6-7 |
| Participant timeline | #13 | Time schedule of enrolment, interventions (including any run-ins and washouts), assessments, and visits for participants. A schematic diagram is highly recommended (see Figure) | Table 1 |
| Sample size | #14 | Estimated number of participants needed to achieve study objectives and how it was determined, including clinical and statistical assumptions supporting any sample size calculations | 11 |
| Recruitment | #15 | Strategies for achieving adequate participant enrolment to reach target sample size | 11 |
| Allocation: | #16a | Method of generating the allocation sequence (eg, | 8 |

For peer review only

| | | | |
|---|---|---|---|
| sequence generation | | computer-generated random numbers), and list of any factors for stratification. To reduce predictability of a random sequence, details of any planned restriction (eg, blocking) should be provided in a separate document that is unavailable to those who enrol participants or assign interventions | |
| Allocation concealment mechanism | #16b | Mechanism of implementing the allocation sequence (eg, central telephone; sequentially numbered, opaque, sealed envelopes), describing any steps to conceal the sequence until interventions are assigned | 8 |
| Allocation: implementation | #16c | Who will generate the allocation sequence, who will enrol participants, and who will assign participants to interventions | 8 |
| Blinding (masking) | #17a | Who will be blinded after assignment to interventions (eg, trial participants, care providers, outcome assessors, data analysts), and how | 10 |
| Blinding (masking): emergency unblinding | #17b | If blinded, circumstances under which unblinding is permissible, and procedure for revealing a participant's allocated intervention during the trial | n/a |
| Data collection plan | #18a | Plans for assessment and collection of outcome, baseline, and other trial data, including any related processes to promote data quality (eg, duplicate measurements, training of assessors) and a description of study instruments (eg, questionnaires, laboratory tests) along with their reliability and validity, if known. Reference to where data collection forms can be found, if not in the protocol | 9-10 |
| Data collection plan: retention | #18b | Plans to promote participant retention and complete follow-up, including list of any outcome data to be collected for participants who discontinue or deviate from intervention protocols | 10 |
| Data management | #19 | Plans for data entry, coding, security, and storage, including any related processes to promote data | 10 |

For peer review only

| | | | |
|---|---|---|---|
| | | quality (eg, double data entry; range checks for data values). Reference to where details of data management procedures can be found, if not in the protocol | |
| Statistics: outcomes | #20a | Statistical methods for analysing primary and secondary outcomes. Reference to where other details of the statistical analysis plan can be found, if not in the protocol | 11-12 |
| Statistics: additional analyses | #20b | Methods for any additional analyses (eg, subgroup and adjusted analyses) | 12 |
| Statistics: analysis population and missing data | #20c | Definition of analysis population relating to protocol non-adherence (eg, as randomised analysis), and any statistical methods to handle missing data (eg, multiple imputation) | 11 |
| Data monitoring: formal committee | #21a | Composition of data monitoring committee (DMC); summary of its role and reporting structure; statement of whether it is independent from the sponsor and competing interests; and reference to where further details about its charter can be found, if not in the protocol. Alternatively, an explanation of why a DMC is not needed | 12 |
| Data monitoring: interim analysis | #21b | Description of any interim analyses and stopping guidelines, including who will have access to these interim results and make the final decision to terminate the trial | 12 |
| Harms | #22 | Plans for collecting, assessing, reporting, and managing solicited and spontaneously reported adverse events and other unintended effects of trial interventions or trial conduct | 12 |
| Auditing | #23 | Frequency and procedures for auditing trial conduct, if any, and whether the process will be independent from investigators and the sponsor | Audits are randomly performed on trials in the institute (Erasmus MC which is the sponsor) |

For peer review only

| | | | |
|---|---|---|---|
| Research ethics approval | #24 | Plans for seeking research ethics committee / institutional review board (REC / IRB) approval | 12 |
| Protocol amendments | #25 | Plans for communicating important protocol modifications (eg, changes to eligibility criteria, outcomes, analyses) to relevant parties (eg, investigators, REC / IRBs, trial participants, trial registries, journals, regulators) | 12 |
| Consent or assent | #26a | Who will obtain informed consent or assent from potential trial participants or authorised surrogates, and how (see Item 32) | 7-8 |
| Consent or assent: ancillary studies | #26b | Additional consent provisions for collection and use of participant data and biological specimens in ancillary studies, if applicable | 8 |
| Confidentiality | #27 | How personal information about potential and enrolled participants will be collected, shared, and maintained in order to protect confidentiality before, during, and after the trial | 10 |
| Declaration of interests | #28 | Financial and other competing interests for principal investigators for the overall trial and each study site | 14 |
| Data access | #29 | Statement of who will have access to the final trial dataset, and disclosure of contractual agreements that limit such access for investigators | 14 |
| Ancillary and post trial care | #30 | Provisions, if any, for ancillary and post-trial care, and for compensation to those who suffer harm from trial participation | 12 |
| Dissemination policy: trial results | #31a | Plans for investigators and sponsor to communicate trial results to participants, healthcare professionals, the public, and other relevant groups (eg, via publication, reporting in results databases, or other data sharing arrangements), including any publication restrictions | 12 |
| Dissemination | #31b | Authorship eligibility guidelines and any intended | 14 |

For peer review only

| | | | |
|---|---|---|---|
| policy: authorship | | use of professional writers | |
| Dissemination policy: reproducible research | #31c | Plans, if any, for granting public access to the full protocol, participant-level dataset, and statistical code | n/a |
| Informed consent materials | #32 | Model consent form and other related documentation given to participants and authorised surrogates | In Dutch |
| Biological specimens | #33 | Plans for collection, laboratory evaluation, and storage of biological specimens for genetic or molecular analysis in the current trial and for future use in ancillary studies, if applicable | 9 |

The SPIRIT checklist is distributed under the terms of the Creative Commons Attribution License CC-BY-ND 3.0. This checklist can be completed online using https://www.goodreports.org/, a tool made by the EQUATOR Network in collaboration with Penelope.ai

For peer review only

BMJ Open

# The CoDiNOS trial protocol: An international randomized controlled trial of intravenous sildenafil versus inhaled nitric oxide for the treatment of pulmonary hypertension in neonates with congenital diaphragmatic hernia.



| | |
|---|---|
| Journal: | *BMJ Open* |
| Manuscript ID | bmjopen-2019-032122.R1 |
| Article Type: | Protocol |
| Date Submitted by the Author: | 05-Aug-2019 |



| | |
|---|---|
| Complete List of Authors: | Cochius - den Otter, Suzan; Erasmus University Rotterdam, Pediatric Intensive Care<br>Schaible, Thomas; University Medical Center, Mannheim<br>Greenough, Anne; Kings College London, Department of Women and Children's Health, School of Life Sciences, Faculty of Life Science and Medicine<br>van Heijst, Arno; Radboudumc, Division of Neonatology, Department of Pediatrics<br>Patel, Neil; Royal Hospital for Children Glasgow, Departement of Neonatology<br>Allegaert, Karel; Erasmus MC Sophia, Pediatrics, Division of Neonatology<br>van Rosmalen, Joost; Erasmus MC, Biostatistics<br>Tibboel, Dick; Erasmus MC-Sophia Children's Hospital, Intensive Care and Pediatric Surgery |



| | |
|---|---|
| <b>Primary Subject Heading</b>: | Paediatrics |
| Secondary Subject Heading: | Evidence based practice, Intensive care, Pharmacology and therapeutics |
| Keywords: | congenital diaphragmatic hernia, sildenafil, nitric oxide, THERAPEUTICS, pulmonary hypertension |
| | |


SCHOLARONE™ Manuscripts

# The CoDiNOS trial protocol: An international randomized controlled trial of intravenous sildenafil versus inhaled nitric oxide for the treatment of pulmonary hypertension in neonates with congenital diaphragmatic hernia.

Suzan CM Cochius – den Otter MD[1], Thomas Schaible MD PhD[2], Anne Greenough MD[3], Arno van Heijst MD PhD[4], Neil Patel MD[5], Karel Allegaert MD PhD[1,6], Joost van Rosmalen PhD[7], Dick Tibboel MD PhD[1] on behalf of the CDH EURO Consortium

[1]Intensive Care and department of Pediatric Surgery, Erasmus MC, Rotterdam, the Netherlands. [2]Department of Neonatology Universitätsmedizin Mannheim, Mannheim, Germany. [3] The Asthma UK Centre in Allergic Mechanisms of Asthma; Women and Children's Health, School of Life Course Sciences, Faculty of Life Sciences and Medicine, King's College London, Denmark Hill, London, United Kingdom.[4] Division of Neonatology, Department of Pediatrics, Radboudumc Amalia Children's Hospital, Nijmegen, The Netherlands. [5]Departement of Neonatology, Royal Hospital for Children, Glasgow, United Kingdom. [6]Department of Development and Regeneration, KU-Leuven, Leuven, Belgium. [7]Department of Biostatistics, Erasmus MC, Rotterdam, The Netherlands.

Corresponding author

Suzan CM Cochius – den Otter

Erasmus MC-Sophia Children's Hospital

Intensive Care Unit

Wytemaweg 80

3015 CN Rotterdam

Tel.: (+31) 10 7031769

Fax: (+31) 10 7037162

s.denotter@erasmusmc.nl

**Word count:** 3004

**Keywords:** Congenital Diaphragmatic Hernia, Pulmonary Hypertension, Therapeutics, Sildenafil, Nitric Oxide



For peer review only

For peer review only - http://bmjopen.bmj.com/site/about/guidelines.xhtml

## Abstract

### Introduction

Congenital diaphragmatic hernia (CDH) is a developmental defect of the diaphragm that impairs normal lung development, causing pulmonary hypertension (PH). PH in CDH newborns is the main determinant for morbidity and mortality. Different therapies are still mainly based on "trial and error". Inhaled nitric oxide (iNO) is often the drug of first choice. However, iNO does not seem to improve mortality. Intravenous (iv) sildenafil has reduced mortality in newborns with PH without CDH, but prospective data in CDH patients are lacking.

### Methods and analysis

In an open label, multicenter, international randomized controlled trial in Europe, Canada and Australia, 330 newborns with CDH and PH are recruited over a four-year period (2018-2022). Patients are randomized for iv sildenafil or iNO. Sildenafil is given in a loading dose of 0.4 mg/kg in 3 hours; followed by continuous infusion of 1.6 mg/kg/day, iNO is dosed at 20 ppm.

Primary outcome is absence of PH on day 14 without pulmonary vasodilator therapy and/or absence of death within the first 28 days of life. Secondary outcome measures include clinical and echocardiographic markers of PH in the first year of life.

We hypothesize that sildenafil gives a 25% reduction in the primary outcome from 68% to 48% on day 14, for which a sample size of 330 patients is needed. An intention-to-treat analysis will be performed. A p-value (two-sided) < 0.05 is considered significant in all analyses.

### Ethics and dissemination

Ethics approval has been granted by the ethics committee in Rotterdam (MEC-2017-324) and the central Committee on Research Involving Human Subjects (NL60229.078.17) in the Netherlands. The principles of the Declaration of Helsinki, the Medical Research Involving Human Subjects Act, and the national rules and regulations on personal data protection will be used. Parental informed consent will be obtained.

### Registration

Trial registration number NTR6982 (Trial NL6796).


For peer review only



For peer review only - http://bmjopen.bmj.com/site/about/guidelines.xhtml

**Article summary**

**Strengths and limitations of this study**

- **The CoDiNOS trial is the first randomized controlled multicenter trial to evaluate the effect of intravenous sildenafil and compare with iNO on pulmonary hypertension in newborns with CDH.**
- **Treatment allocation is not blinded in the trial. This is not feasible because of variability in iNO equipment and gas mixtures use. Instead, the researchers who analyze the echocardiography to evaluate PH will be blinded to the treatment.**
- **The primary outcome, PH, will be measured using echocardiography instead of just clinical parameters often used in newborns**
- **There is no non-intervention group, as it is common practice in the centers of the CDH EURO Consortium to give iNO; hence, it is considered unethical to withhold treatment for one group.**
- **Long term follow up of 12 months will give more insight in the course of PH in infants**

For peer review only



For peer review only - http://bmjopen.bmj.com/site/about/guidelines.xhtml

**Introduction:**

Congenital diaphragmatic hernia (CDH) is a severe developmental defect of the diaphragm with an incidence of approximately 1 in 3000 live births and a mortality of 27% [1]. Because of this defect, the abdominal organs herniate into the chest causing pulmonary hypoplasia and abnormal pulmonary vasculature growth, resulting in pulmonary hypertension (PH) [2]. In adults and children, PH is defined as mean pulmonary artery pressure (mPAP) exceeding 25 mmHg with a pulmonary capillary wedge pressure of minimal 15 mmHg [3].

The normal pulmonary vascular transition of the neonate takes around two months to achieve these low values of mPAP. During fetal life, there is high resistance in the pulmonary circulation which results in most of the blood flow to bypass the lungs through the ductus arteriosus and oval foramen. Immediately after birth, the pulmonary vascular resistance drops and the blood flow to the lungs significantly increases [4]. In contrast, the pulmonary vascular resistance often does not drop adequately in children with CDH due to a decreased vascular bed associated with lung hypoplasia, and an altered development of the pulmonary vasculature with excessive muscularization of the arterioles, with increased thickness of the arterial media and adventitia. Although the presence of lung hypoplasia can be predicted with prenatal parameters, reliable predictors for PH in CDH patients are lacking [5]. The incidence of PH in CDH patients is 68-79% and causes considerable morbidity and mortality [1, 2, 6]. Therapy in newborns with PH , such as inhaled nitric oxide (iNO) and sildenafil, has improved outcomes in general. However, trials in infants with CDH are sparse.

Inhaled nitric oxide (iNO) diffuses rapidly across the alveolar-capillary membrane into the smooth muscle of pulmonary vessels to activate soluble guanylate cyclase. This enzyme mediates many of the biological effects of iNO, and is responsible for the conversion of GTP to cGMP. The increase of intracellular cGMP relaxes smooth muscles via several mechanisms. iNO also causes bronchodilation and has anti-inflammatory and anti-proliferative effects [7]. In term and near term infants with persistent pulmonary hypertension of the newborn (PPHN), iNO decreases the median duration of mechanical ventilation and reduces the need for extracorporeal membrane oxygenation (ECMO). However, in the two available randomized controlled trials (RCT) with a small number of patients with CDH, mortality did not improve and more ECMO treatment was needed despite short-term improved of oxygenation in some treated patients [8, 9]. In the centers of the CDH EURO Consortium, iNO is standard of care in infants with CDH and PH although the positive pharmacodynamic effects in these infants are less convincing then in infants with PPHN [6, 10]. The pathophysiological mechanism of this difference is not understood. In resource poor settings iNO is often unavailable. In



For peer review only - http://bmjopen.bmj.com/site/about/guidelines.xhtml

the search to find another treatment option, trials to evaluate the effect of sildenafil in newborns with PPHN have been conducted [11].

Sildenafil is a selective phosphodiesterase type 5 (PDE5) inhibitor. PDE5 is an enzyme that specifically degrades cGMP. Sildenafil inhibits PDE5, increasing cGMP and NO-mediated vasodilatation of the smooth muscles in arteries. Only five RCTs have been performed in newborns, all non-CDH patients with PPHN. Four of these studies showed a decrease in oxygenation index (OI) and mortality in a setting where iNO was not available, while one trial showed no additional benefit of sildenafil when added to iNO [11]. Although sildenafil is increasingly used in CDH patients, only retrospective data are available [12]. A decrease in pulmonary vascular resistance index and an increase in cardiac output were found in a small group of oral sildenafil-treated infants with CDH refractory to iNO [13]. Intravenous sildenafil improved OI and reversed the right-to-left shunt ratio over the PDA, but it also increased the need for inotropic support [14, 15]. However, its effect on outcome is unknown.

We hypothesize that intravenous sildenafil is superior to iNO. iNO is the therapy of first choice in most centers despite the lack of evidence, and sildenafil is the most promising drug for the treatment of PH in CDH patients and is increasingly being used [6, 12, 16]. However, no studies have been performed comparing iNO with intravenous sildenafil in newborns with CDH and PH or PH alone. Based on the current knowledge, there is equipoise for both treatment modalities.

For peer review only



For peer review only - http://bmjopen.bmj.com/site/about/guidelines.xhtml

**Methods and analysis:**

**Design**

The CoDiNOS trial is a prospective, multicenter, international randomized controlled trial conducted in high volume pediatric surgical centers in Europe, Canada and Australia. The members of the CDH Euro Consortium participating in the trial are listed in the Appendix.

**Objectives**

The primary objective of the study is to determine whether the incidence of PH is lower in CDH patients treated with intravenous sildenafil than in patients treated with iNO, with the primary outcome defined as the absence of PH on echocardiography on day 14 without pulmonary vasodilator therapy and without treatment failure and/or death within the first 28 days after birth. PH is defined as systolic pulmonary arterial pressure> 2/3 systolic systemic pressure and/or right ventricular (RV) dilatation/septal displacement and RV dysfunction +/- left ventricular dysfunction.

The secondary outcomes are:

(1) change in OI after 12 and 24 hours of therapy

(2) overall mortality

(3) the incidence of treatment failure which is defined as:

- inability to maintain preductal saturations above 85% (± 7 kPa or 52 mmHg) or postductal saturations above 70% (±5.3 kPa or 40 mmHg)
- and/or increase in $CO_2$ > 70 mmHg (9.3 kPa) despite optimization of ventilator management
- and/or inadequate oxygen delivery with metabolic acidosis defined as lactate ≥ 5 mmol/l and pH < 7.15 and/or hypotension resistant to fluid therapy and adequate inotropic support resulting in a urine output < 0.5 ml/kg/hour
- and/or lactate ≥ 5 mmol/l and pH < 7.15
- and/or OI consistently ≥ 40

(4) time on intervention drug, defined as intervention drug free days after initiation of the intervention, calculated on day 14

(5) need for ECMO



For peer review only - http://bmjopen.bmj.com/site/about/guidelines.xhtml

(6) ventilator free days on day 28

(7) the use of drugs for PH treatment during the hospital admission

(8) the use of pulmonary and/or cardiac medication at discharge and its total duration of administration

(9) short-term and long-term PH on echocardiography at 24 hours, 28 days/discharge and 6 and 12 months

(10) the incidence of chronic lung disease

(11) the development of neurological abnormalities evaluated with ultrasound of the brain before the start of the trial, after surgery and before discharge

(12) the external validation of the sildenafil PKPD model for the pharmacokinetics and the pharmacodynamic effects of sildenafil

Safety outcomes include adverse events due to the study drugs and the vasoactive-inotropic support score (VIS).

**Patients**

Infants diagnosed with CDH who have PH in the first week after birth, are eligible for the trial if born at or after a gestational age of 34 weeks. The diagnosis of PH is defined as at least two of the following four criteria: (I) systolic pulmonary arterial pressure> 2/3 systolic systemic pressure estimated by echocardiography. (II) RV dilatation/septal displacement, RV dysfunction +/- left ventricular dysfunction. (III) Pre-post ductal $SpO_2$ difference > 10%. (IV) OI >20. Exclusion criteria are a severe chromosomal anomaly which may imply a decision to stop or not to start life-saving medical treatment, severe cardiac anomaly expected to need corrective surgery in the first 60 days of life, renal anomalies associated with oligohydramnios, severe orthopedic and skeletal deformities, which are likely to influence thoracic, and / or lung development and severe anomalies of the central nervous system. Patients who are born in another center and transported with iNO are also excluded from the trial. Patients who received fetal interventions (trachea balloon placement) are not excluded.

Following antenatal diagnosis, the parents are counselled and informed about the study by the clinician or research coordinator. Also, they receive a patient information letter and an informed consent form . If the patient is not born in a participating center or the diagnosis of CDH was not known, parents are counselled after the diagnosis of CDH and are informed about the study. Also,



For peer review only - http://bmjopen.bmj.com/site/about/guidelines.xhtml

they receive written information and an informed consent form. This informed consent form contains consent for the trial and for collection of data and material for future research.

For the development of the protocol the SPIRIT reporting guidelines have been used [17]. This publication is based on protocol version 4, June 13th 2018.

**Patient and public involvement**

Patients and the public were not involved in the development of the trial protocol. However, CDH UK Sparks, as a parent organization, has assessed and commented on the protocol and as provided start-up funding as also mentioned in the funding statement. This organization is and will be regularly informed on progress and results of the trial.

**Study procedures**

*Baseline assessment*

Antenatal ultrasound data about the characteristics of the CDH are collected. These data include the observed/expected lung-head ratio, position of the liver and stomach and the amniotic fluid index. An MRI or an ultrasound is performed depending on local experience and possibilities. If an MRI is performed, the observed/expected fetal lung volume will be calculated. Also data on prenatal interventions are collected. In all mothers, a planned vaginal or caesarean delivery is pursued.

*Randomization, intervention and blinding*

When the patient meets the inclusion criteria, the physician logs in to the web based program, which randomizes the patient with a computer-generated randomization list, made by the independent statistician of the Data Safety and Monitoring Board. Blocked randomization, with variable block sizes and stratification by center, is used to achieve equal distribution of the two interventions among the participants.

Postnatally, infants are treated according to a standardized protocol for patients with CDH, which is implemented in all participating centers. This protocol was developed with the available evidence and consensus between the participating centers and was updated in June 2016 [10, 16]. If the patient is diagnosed with PH in the first week of life, the patient will be allocated to one of the two study drugs (figure 1). iNO is provided by a tank connected to a ventilator. Different devices are used in different centers. Some centers use integrated systems, making it impossible to disconnect the iNO tank and replace it with another gas to facilitate a blinded intervention. Therefore, the study is open label. iNO is given with a starting dose of 20 ppm, which is the maximum dose [18, 19]. Sildenafil is given intravenously, using a loading dose of 0.4mg/kg in 3 hours, followed by continuous



For peer review only - http://bmjopen.bmj.com/site/about/guidelines.xhtml

infusion of 1.6mg/kg/day [20, 21]. To wean the study drugs a standard protocol is followed (figure 2). The allocated drugs will be restarted as per protocol if criteria for its use are met again before the age of 14 days. To further standardize care, an inotropic support flow chart is included in the study protocol (figure 3). After day 14 treatment of PH will be at the discretion of the local medical team and the study drug can be changed to, for instance, sildenafil orally. The use of bosentan, milrinone and prostin next to the study treatment is allowed. The use of bosentan as add on therapy is allowed and is considered as PH treatment on day 14.The intervention will be prematurely stopped when the patient meets one or more of the defined failure criteria, described in point three of the secondary outcomes. Further treatment will then be at the discretion of the medical team and will be according to the standardized protocol[16]. INO and sildenafil can both be given outside the study protocol. An ECMO-procedure may then be started in centers where ECMO is available. Data of all patients are used in the intention-to-treat analysis.

*Follow up*

After day 14, additional clinical data, such as time on ventilator support (days) and the use of drugs for the treatment of PH, are collected to answer the secondary outcome questions. Also, echocardiographic measurements are taken at 6 and 12 months to evaluate the presence of chronic PH (table 1)

Table 1 Procedures and measurements

| | Day 0-7 before start thera-py | 3 hrs after start silde-nafil | 12hrs after start | 8 am after start | 24hrs after start | Day of surgery, pre-opera-tively | Day after sur-gery | Day of ECMO, pre-cannula-tion | 8 am after start ECMO | Day 14 | Day 28 / before dis-charge | Day 56 | 6 mnth | 12 mnth |
|---|---|---|---|---|---|---|---|---|---|---|---|---|---|---|
| Echocardio-graphy | X | | | | X | | | | | X | X | | X | X |
| Calculation OI | X | | X | | X | | | | | | | | | |
| Calculation VIS score | X | | X | | X | | | | | | | | | |
| Blood sample | X | | | X | | X | X | X | X | X | | | | |
| Tracheal aspirate | X | | | X | | X | X | X | X | X | | | | |
| Urine sample | X | | | X | | X | X | X | X | X | | | | |
| Severity of CLD | | | | | | | | | | | X | X | | |
| Ultrasound brain | X | | | | | | X | | | | | | | |
| Sildenafil plasma | | X | | X | | X | X | | X | | | | | |



For peer review only

For peer review only - http://bmjopen.bmj.com/site/about/guidelines.xhtml

| | | | | | | | | | | | | | | |
|---|---|---|---|---|---|---|---|---|---|---|---|---|---|---|
| level | | | | | | | | | | | | | | |

OI: oxygenation index; VIS score: vasoactive-inotropic support score; CLD: chronic lung disease; ECMO: extracorporeal membrane oxygenation

*Data collection*

Echocardiography parameters are measured by local physicians, centrally collected and reviewed by two blinded independent physicians to reduce inter-observer variation. Demographic and neonatal characteristics as well as data on the clinical course of all patients are entered in a password protected web-based database in Rotterdam (OpenClinica). Upon request the collected data will be available. All centers will keep a logbook of the number of non-participants, including the reasons for not participating. Study documents are securely stored at each study site for 15 years.

*Laboratory testing*

Blood, urine and tracheal samples are collected in most centers during the trial. Blood samples are collected before the start of the study and at different time points until day 14. Some samples will be used to externally validate a NONMEM prediction model for sildenafil. The other samples will be used in future research on biomarkers to predict severity and outcome of PH in CDH patients. The samples are centrifuged for 6 minutes at 3000 rpm [22]. Thereafter, the plasma is removed and stored at –20 degrees Celsius or colder. The total amount of blood taken is maximal 2.5 % of the circulating volume. Blood sampling will only be done if a central or peripheral line is still present and/or in combination with routine laboratory measurements. This way blood sampling is a minimal burden for the patient.

Tracheal aspirate for proteomic analysis is also collected at different time points during routine tracheal suctioning in ventilated patients. Protein profiling with proteomics is used to identify specific groups of proteins that are involved in the pathogenesis of PH. The tracheal aspirates is centrifuged for 6 minutes at 3000 rpm and stored at –80 degrees Celsius [23].

Also, 8-hour urine is collected at different time points. Two samples of 5 ml are taken and stored at –20 degrees Celsius or colder.

*Withdrawal of participants*

Parents may decide to withdraw from the study at any time without any consequences. The investigator can decide to withdraw a patient from the study for urgent medical reasons. In some cases, there may be exclusion criteria, which were not known before randomization. If this is the case, the patient will be withdrawn from the study after contacting the study coordinator. With

For peer review only

consent of the parents data will still be collected, stored and analyzed to perform an intention-to-treat analysis. These children will be treated according to standard practice [10, 16].

**Sample size calculation**

We powered our study using PH at day 14 as primary outcome. Lusk et al. showed that PH, defined as >2/3 systemic blood pressure measured on echocardiography, in CDH patients on day 14 has a positive predictive value of 0.8 for death, death or ventilation, and death or ventilator support. PH on day 14 is observed in 64% of CDH patients [24].

Even dough the definition is not the same, we assume a similar outcome percentage of 64% for failing the primary outcome in our trial, the absence of PH on day 14 without pulmonary vasodilator therapy and/or absence of death within the first 28 days of life, in the iNO group. For a 25% relative reduction to 48%, a sample size of 300 patients (150 patients per group) is needed to obtain a power of 80%. This will match a number needed to treat of 6.25. Taking missing data and the effects of correction for covariates into account, we adjust this sample size to 330 patients. In the collaborating centers 550 patients will be born in three years. Based on our earlier trial (VICI trial) we expect to have an inclusion rate of 60%. Therefore, the inclusion of 330 patients should be reached in three years.

**Data analysis**

The patients will be analyzed according to the group they are randomized to (intention-to-treat analysis). A p-value (two-sided) < 0.05 is considered significant in all analyses. The primary endpoint PH will be analyzed using multiple logistic regression with randomization arm, center, observed/expected head-lung ratio, position of the liver, side of the defect, defect size and ventilation modality as independent variables [25]. If necessary, multiple imputation using the fully conditional specification method will be used to account for missing data in the independent variables. We will perform a sensitivity analyses with adjustment for the use of prostin and milrinone, to account for the effects of these vasodilators on PH.

The following analyses will be performed for the secondary outcomes. The distribution of VIS score in all study participants will be compared between t=0 and t=12 hours after initiation of drug administration using a Wilcoxon signed rank test. The distribution of changes in OI and VIS score from t=0 to t=12 and t=24 hours will be compared between the randomization groups with a Mann-Whitney test. The overall mortality in the first year of life will be compared between the



For peer review only - http://bmjopen.bmj.com/site/about/guidelines.xhtml

randomization groups with Kaplan-Meier curves and the log-rank test. The number of treatment failures, the need for ECMO (in ECMO centers), and the need for medication for PH or chronic lung disease at discharge, and during the first year of life, will be compared between randomization groups with chi-square tests. The number of study drug free days at day 14, the number of ventilation-free days until day 28, the fraction of days with need for medical treatment (excluding the study drug) for PH during the hospital admission, and the severity of chronic lung disease using the Bancalari definition, will be compared between randomization groups with Mann-Whitney tests. Deaths will be counted as the worst outcome in these analyses, in accordance with the intention-to-treat principle. The presence of PH at 28 days/discharge, 6 and 12 months according to the echocardiographic parameters will be compared between randomization groups with a chi-square test.

To externally validate the pharmacokinetic model of sildenafil and it active metabolite (built in NONMEM) Normalized prediction distribution errors (NPDE) and Visual Predictive Check (VPC) will be used. Furthermore, the model will be used to predict the drug concentrations from the new data set using simulations, in which we expect that the difference will be less than 20%. To find a relationship between the concentration of sildenafil, its active metabolite and the clinical effects, such as OI, VIS score and echocardiography measures, a Mann-Whitney or T test will be used.

**Safety reporting and trial oversight**

All severe adverse events (SAEs) and suspected unexpected serious adverse reactions (SUSARs) are reported from the enrolment until 12 month follow-up. Persistent or significant disability or incapacity that was not expected with the given O/E LHR is evaluated as an SAE. An elective hospital admission is not a SAE. All SAEs and SUSARs are reported to the approving ethics committees in accordance with their requirements. We will report the SAEs and SUSARs that result in death or are life threatening within 7 days of first knowledge. All other SAEs and SUSARs will be reported within a period of maximum 15 days. Once a year throughout the clinical trial, we will submit a safety report to the approving ethics committees and competent authorities of the countries involved.

The trial will be monitored by qualified, independent monitors. The trial is classified as a trial with moderate risk and a specific monitoring plan is in place.

The data safety monitoring board will monitor the incidence of mortality on a continuous basis. If at some point a large difference in mortality between the two treatment groups is noticed, the data safety monitoring board may recommend ending the study.

Insurance will cover compensation to patients who suffer harm from trial participation.



For peer review only - http://bmjopen.bmj.com/site/about/guidelines.xhtml

**Ethics and dissemination**

Ethics approval has been granted by the local ethics committee in Rotterdam (MEC-2017-324) and by the central Committee on Research Involving Human Subjects (NL60229.078.17) in the Netherlands. Important amendments will be communicated to all relevant parties. The study will be conducted according to the principles of the Declaration of Helsinki, in accordance with the Medical Research Involving Human Subjects Act, and national rules and regulations on personal data protection. Parental informed consent will be obtained. The results of this study will be disseminated via peer-reviewed publications and implemented in the international guidelines for the treatment of newborns with CDH.

For peer review only



For peer review only - http://bmjopen.bmj.com/site/about/guidelines.xhtml

**Literature**


1. Snoek, K.G., et al., *Conventional Mechanical Ventilation Versus High-frequency Oscillatory Ventilation for Congenital Diaphragmatic Hernia: A Randomized Clinical Trial (The VICI-trial).* Ann Surg, 2015.
2. Robinson, P.D. and D.A. Fitzgerald, *Congenital diaphragmatic hernia.* Paediatr Respir Rev, 2007. **8**(4): p. 323-34; quiz 334-5.
3. Galie, N., et al., *Guidelines for the diagnosis and treatment of pulmonary hypertension: the Task Force for the Diagnosis and Treatment of Pulmonary Hypertension of the European Society of Cardiology (ESC) and the European Respiratory Society (ERS), endorsed by the International Society of Heart and Lung Transplantation (ISHLT).* Eur Heart J, 2009. **30**(20): p. 2493-537.
4. Vali, P. and S. Lakshminrusimha, *The Fetus Can Teach Us: Oxygen and the Pulmonary Vasculature.* Children (Basel), 2017. **4**(8).
5. Russo, F.M., et al., *Lung size and liver herniation predict need for extracorporeal membrane oxygenation but not pulmonary hypertension in isolated congenital diaphragmatic hernia: systematic review and meta-analysis.* Ultrasound Obstet Gynecol, 2017. **49**(6): p. 704-713.
6. Putnam, L.R., et al., *Evaluation of Variability in Inhaled Nitric Oxide Use and Pulmonary Hypertension in Patients With Congenital Diaphragmatic Hernia.* JAMA Pediatr, 2016.
7. Ichinose, F., J.D. Roberts, Jr., and W.M. Zapol, *Inhaled nitric oxide: a selective pulmonary vasodilator: current uses and therapeutic potential.* Circulation, 2004. **109**(25): p. 3106-11.
8. *Inhaled nitric oxide and hypoxic respiratory failure in infants with congenital diaphragmatic hernia. The Neonatal Inhaled Nitric Oxide Study Group (NINOS).* Pediatrics, 1997. **99**(6): p. 838-45.
9. Clark, R.H., et al., *Low-dose nitric oxide therapy for persistent pulmonary hypertension of the newborn. Clinical Inhaled Nitric Oxide Research Group.* N Engl J Med, 2000. **342**(7): p. 469-74.
10. Reiss, I., et al., *Standardized postnatal management of infants with congenital diaphragmatic hernia in Europe: the CDH EURO Consortium consensus.* Neonatology, 2010. **98**(4): p. 354-64.
11. Kelly, L.E., A. Ohlsson, and P.S. Shah, *Sildenafil for pulmonary hypertension in neonates.* Cochrane Database Syst Rev, 2017. **8**: p. CD005494.
12. Lakshminrusimha, S., et al., *Milrinone in congenital diaphragmatic hernia - a randomized pilot trial: study protocol, review of literature and survey of current practices.* Matern Health Neonatol Perinatol, 2017. **3**: p. 27.
13. Noori, S., et al., *Cardiovascular effects of sildenafil in neonates and infants with congenital diaphragmatic hernia and pulmonary hypertension.* Neonatology, 2007. **91**(2): p. 92-100.
14. Bialkowski, A., F. Moenkemeyer, and N. Patel, *Intravenous sildenafil in the management of pulmonary hypertension associated with congenital diaphragmatic hernia.* Eur J Pediatr Surg, 2015. **25**(2): p. 171-6.
15. Kipfmueller, F., et al., *Continuous intravenous sildenafil as an early treatment in neonates with congenital diaphragmatic hernia.* Pediatr Pulmonol, 2018.
16. Snoek, K.G., et al., *Standardized Postnatal Management of Infants with Congenital Diaphragmatic Hernia in Europe: The CDH EURO Consortium Consensus - 2015 Update.* Neonatology, 2016. **110**(1): p. 66-74.
17. Chan, A.W., et al., *SPIRIT 2013 statement: defining standard protocol items for clinical trials.* Ann Intern Med, 2013. **158**(3): p. 200-7.
18. Tworetzky, W., et al., *Inhaled nitric oxide in neonates with persistent pulmonary hypertension.* Lancet, 2001. **357**(9250): p. 118-20.
19. Barrington, K.J., et al., *Nitric oxide for respiratory failure in infants born at or near term.* Cochrane Database Syst Rev, 2017. **1**: p. CD000399.




For peer review only - http://bmjopen.bmj.com/site/about/guidelines.xhtml

20. Steinhorn, R.H., et al., *Intravenous sildenafil in the treatment of neonates with persistent pulmonary hypertension.* J Pediatr, 2009. **155**(6): p. 841-847 e1.
21. Mukherjee, A., et al., *Population pharmacokinetics of sildenafil in term neonates: evidence of rapid maturation of metabolic clearance in the early postnatal period.* Clin Pharmacol Ther, 2009. **85**(1): p. 56-63.
22. Snoek, K.G., et al., *High-Sensitivity Troponin T and N-Terminal Pro-Brain Natriuretic Peptide in Prediction of Outcome in Congenital Diaphragmatic Hernia: Results from a Multicenter, Randomized Controlled Trial.* J Pediatr, 2016. **173**: p. 245-249 e4.
23. Bowler, R.P., M.C. Ellison, and N. Reisdorph, *Proteomics in pulmonary medicine.* Chest, 2006. **130**(2): p. 567-74.
24. Lusk, L.A., et al., *Persistence of pulmonary hypertension by echocardiography predicts short-term outcomes in congenital diaphragmatic hernia.* J Pediatr, 2015. **166**(2): p. 251-6 e1.
25. Lally, K.P., et al., *Standardized reporting for congenital diaphragmatic hernia--an international consensus.* J Pediatr Surg, 2013. **48**(12): p. 2408-15.

**Author contributions**

All investigators of the Consortium described below, have contributed to the design of the trial protocol and have approved this version for submission. Coordinating investigator S Cochius – den Otter and Prof D Tibboel are responsible for all aspects of the study conduct, practically study oversight, recruitment, training of the participating hospitals, reporting of the SAEs and SUSARs, outcome assessment and data management. Prof D Tibboel, Prof K Allegaert, Dr T Schaible, Dr A van Heijst, Dr A Greenough and Dr N Patel are responsible for study oversight. J van Rosmalen has contributed to statistical methods and will be involved in interpretation of the results. S Cochius- den Otter will lead the dissemination and translation of results with the contribution of all investigators of the CDH EURO Consortium. Also all members will have authority over the data.

**Funding statement**

This work was supported by CDH UK Sparks grant number 16EMC01 and by Stichting Sophia Kinderziekenhuis Fonds grant number S17-19.

**Competing interests**

None

**Data sharing statement:**

Deidentified individual participant data will be made available, in addition to the statistical analysis plan. The data will be made available upon publication to researchers who provide a methodologically sound proposal for use in achieving the goals of the approved proposal. Every center will make data available for sharing after consultation of the PI of that center. Requests can be send to Dick Tibboel (d.tibboel@erasmusmc.nl)

**Figure 1** Trial flow chart



For peer review only - http://bmjopen.bmj.com/site/about/guidelines.xhtml

Flow chart showing the steps of the trial, from birth until 12 months. CDH: congenital diaphragmatic hernia; SAE: serious adverse event; SUSAR: suspected unexpected serious adverse event

**Figure 2** Protocol to wean study drug

Flow chart showing the protocol to wean off inhaled nitric oxide or intravenous sildenafil. iNO: inhaled nitric oxide; ppm: parts per million

**Figure 3** Treatment flow chart of systemic hypotension

Flow chart that is added to the treatment protocol, showing the treatment plan for systemic hypotension. VA ECMO: veno-arterial extracorporeal membrane oxygenation

For peer review only

Figure 1 Trial flow chart

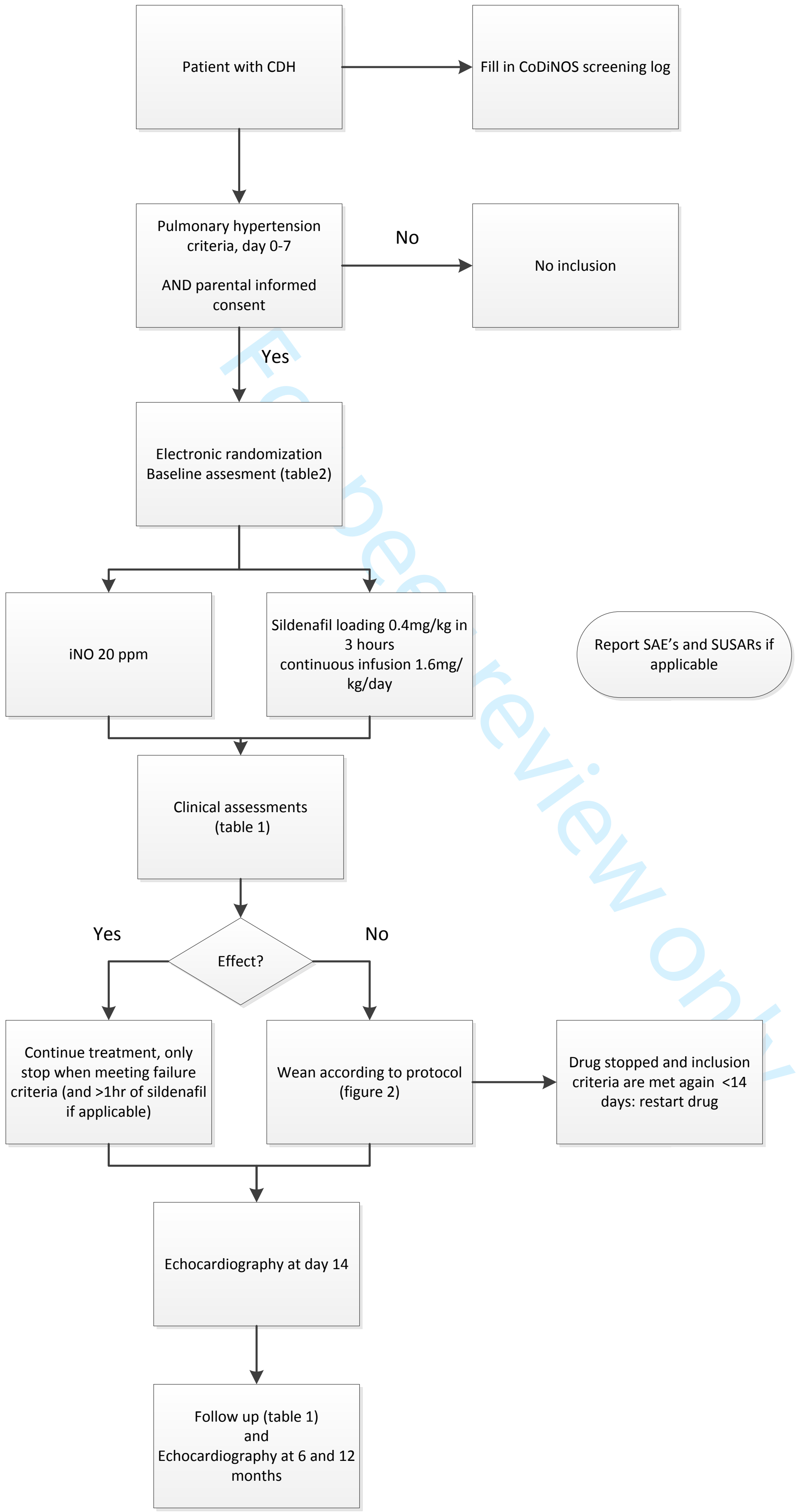


CDH: congenital diaphragmatic hernia; SAE: serious adverse event; SUSAR: suspected unexpected serious adverse event

Figure 2 Protocol to wean study drug

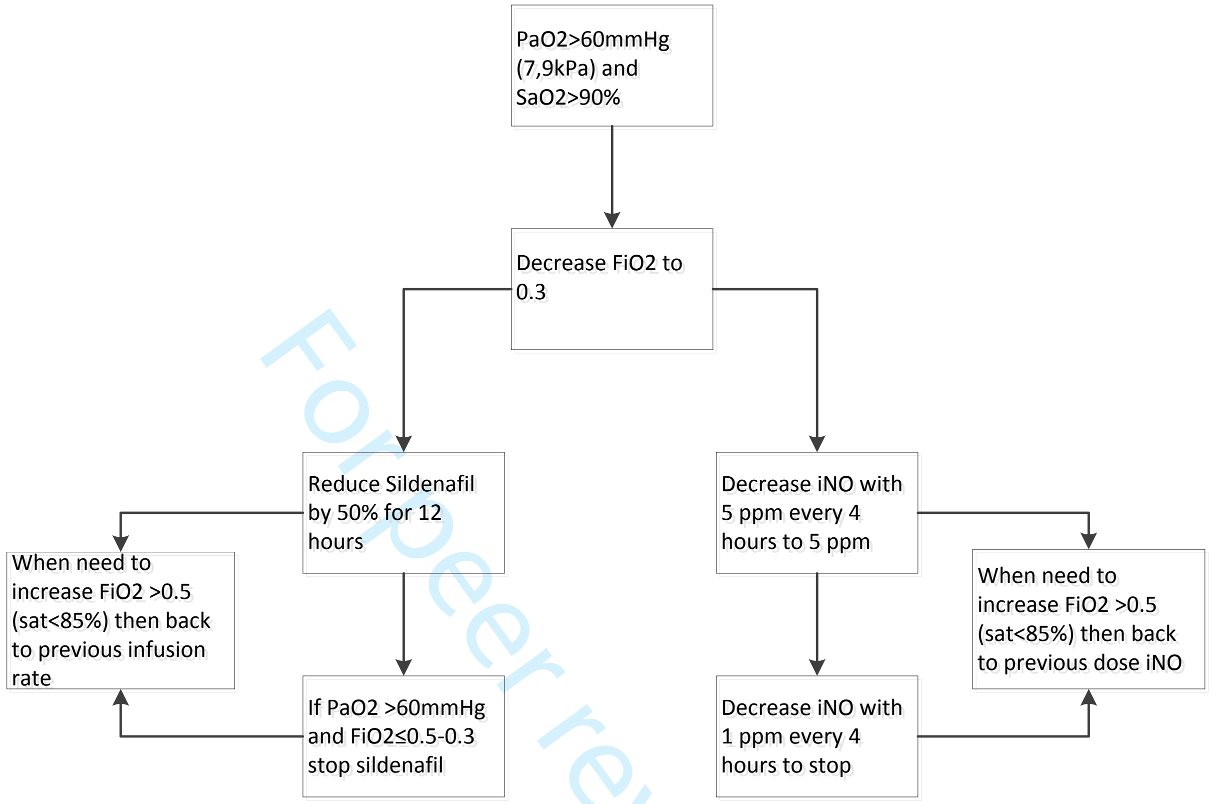


For peer review only

Figure 3: Treatment flow chart of systemic hypotension

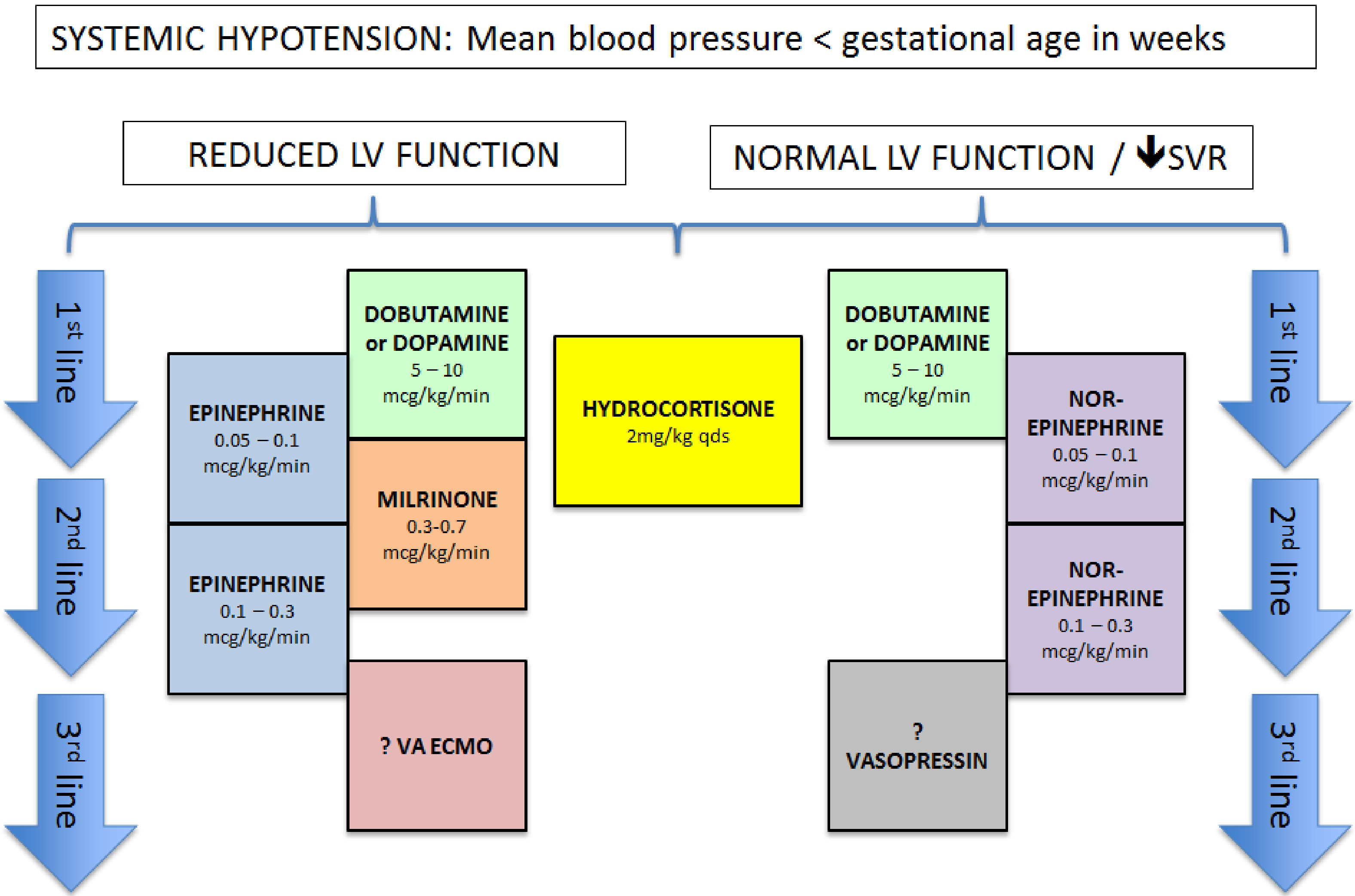

**Appendix:**

CDH Euro Consortium:

Germany: Florian Kipfmueller, Department of Neonatology and Pediatric Critical Care Medicine, University Children's Hospital, Bonn. Spain: Maria Dolores Elorza, Ana Sanchez, Neonatology Department, Leopoldo Martinez, Pediatric Surgery Department, Carlos Labrandero, Viviana Arreo, Pediatric Cardiology Division, Hospital Universitario La Paz, Madrid. Africa Pertierra Cortada, Jordi Clotet Caba Neonatology Department, Hospital Sant Joan de Déu, Barcelona. Marta Aguar, Ana Gimeno, Raquel Escrig, Division of Neonatology, University and Polytechnic Hospital La Fe Valencia. Italy: Irma Capolupo, Pietro Bagolan, Department of Medical and Surgical Neonatology, Bambino Gesu' Children's Hospital, Rome. Fabrizio Ciralli, Genny Raffaeli, Giacomo Cavallaro, Valentina Condò, Fondazione IRCCS Ca' Granda Ospedale Maggiore Policlinico, NICU, University of Milan, Department of Clinical Sciences and Community Health. United Kingdom: United Kingdom - Paul D. Losty Department Of Paediatric Surgery, Division of Child Health, Alder Hey Children's Hospital NHS Foundation Trust, University of Liverpool , Marie Horan, Paediatric Intensive Care Alder Hey Children's Hospital NHS Foundation Trust, University of Liverpool. Nimish V. Subhedar, NICU, Liverpool Women's Hospital, Liverpool. Yogen Singh, Department of Neonatology, Cambridge University Hospitals NHS Foundation trust, Cambridge. Emma E. Williams, The Asthma UK Centre in Allergic Mechanisms of Asthma; Women and Children's Health, School of Life Course Sciences, Faculty of Life Sciences and Medicine, King's College London, Denmark Hill, London. Theodore Dassios, Ravindra Bhat, King's College Hospital NHS Foundation Trust, London. Austria: Jennifer B. Brandt, Alexandra Kreissl, Angelika Berger, Department of Pediatrics and Adolescent Medicine, Division of Neonatology, Pediatric Intensive Care Medicine and Neuropediatrics, Medical University of Vienna. Berndt Urlesberger, Division of Neonatology, Department of Pediatrics and Adolescent Medicine, Medical University of Graz. Sweden: Carmen Mesas Burgos, Björn Frenckner, Department of Pediatric Surgery, Björn Larrson, Pediatric Intensive Care Unit, Karolinska University Hospital, Stockholm. Portugal: Carla Pinto, Serviço de Cuidados Intensivos Pediátricos, Hospital Pediátrico, Centro Hospitalar e Universitário de Coimbra, Coimbra. Joana Saldaha, Department of Neonatology, Hospital de Santa Maria, Lisbon. Belgium: Anne Debeer, Anne Smits, Neonatology, University Hospitals Leuven, Leuven. Norway: Ragnhild Emblem, Department of Pediatric Surgery, Oslo University Hospital, Oslo. Canada: Richard Keijzer, Department of Surgery, Yassar Elsayed, Department of Neonatology, Pediatrics and Child Health, University of Manitoba and Children's Hospital Research Institute of Manitoba. Australia: David Tingay, Department of Neonatology, Royal Children's Hospital, Melbourne, Australia. The Netherlands: Ulrike Kraemer, Intensive Care and department of Pediatric Surgery, Erasmus MC, Rotterdam.

For peer review only

# Reporting checklist for protocol of a clinical trial.

Based on the SPIRIT guidelines.

## Instructions to authors

Complete this checklist by entering the page numbers from your manuscript where readers will find each of the items listed below.

Your article may not currently address all the items on the checklist. Please modify your text to include the missing information. If you are certain that an item does not apply, please write "n/a" and provide a short explanation.

Upload your completed checklist as an extra file when you submit to a journal.

In your methods section, say that you used the SPIRIT reporting guidelines, and cite them as:

Chan A-W, Tetzlaff JM, Altman DG, Laupacis A, Gøtzsche PC, Krleža-Jerić K, Hróbjartsson A, Mann H, Dickersin K, Berlin J, Doré C, Parulekar W, Summerskill W, Groves T, Schulz K, Sox H, Rockhold FW, Rennie D, Moher D. SPIRIT 2013 Statement: Defining standard protocol items for clinical trials. Ann Intern Med. 2013;158(3):200-207

| | | Reporting Item | Page Number |
|---|---|---|---|
| Title | #1 | Descriptive title identifying the study design, population, interventions, and, if applicable, trial acronym | 1 |
| Trial registration | #2a | Trial identifier and registry name. If not yet registered, name of intended registry | 2 |
| Trial registration: data set | #2b | All items from the World Health Organization Trial Registration Data Set | 8 |
| Protocol version | #3 | Date and version identifier | 8 |
| Funding | #4 | Sources and types of financial, material, and other support | 14 |
| Roles and responsibilities: contributorship | #5a | Names, affiliations, and roles of protocol contributors | 14 and appendix |
| Roles and | #5b | Name and contact information for the trial sponsor | 1 |

For peer review only

| | | | |
|---|---|---|---|
| responsibilities: sponsor contact information | | | |
| Roles and responsibilities: sponsor and funder | #5c | Role of study sponsor and funders, if any, in study design; collection, management, analysis, and interpretation of data; writing of the report; and the decision to submit the report for publication, including whether they will have ultimate authority over any of these activities | 14 |
| Roles and responsibilities: committees | #5d | Composition, roles, and responsibilities of the coordinating centre, steering committee, endpoint adjudication committee, data management team, and other individuals or groups overseeing the trial, if applicable (see Item 21a for data monitoring committee) | 14 |
| Background and rationale | #6a | Description of research question and justification for undertaking the trial, including summary of relevant studies (published and unpublished) examining benefits and harms for each intervention | 4-5 |
| Background and rationale: choice of comparators | #6b | Explanation for choice of comparators | 4-5 |
| Objectives | #7 | Specific objectives or hypotheses | 6 |
| Trial design | #8 | Description of trial design including type of trial (eg, parallel group, crossover, factorial, single group), allocation ratio, and framework (eg, superiority, equivalence, non-inferiority, exploratory) | 6 |
| Study setting | #9 | Description of study settings (eg, community clinic, academic hospital) and list of countries where data will be collected. Reference to where list of study sites can be obtained | 6, 14, appendix |
| Eligibility criteria | #10 | Inclusion and exclusion criteria for participants. If applicable, eligibility criteria for study centres and | 7 |

For peer review only

| | | | |
|---|---|---|---|
| | | individuals who will perform the interventions (eg, surgeons, psychotherapists) | |
| Interventions: description | #11a | Interventions for each group with sufficient detail to allow replication, including how and when they will be administered | 8 |
| Interventions: modifications | #11b | Criteria for discontinuing or modifying allocated interventions for a given trial participant (eg, drug dose change in response to harms, participant request, or improving / worsening disease) | 9 |
| Interventions: adherance | #11c | Strategies to improve adherence to intervention protocols, and any procedures for monitoring adherence (eg, drug tablet return; laboratory tests) | n/a |
| Interventions: concomitant care | #11d | Relevant concomitant care and interventions that are permitted or prohibited during the trial | 8 |
| Outcomes | #12 | Primary, secondary, and other outcomes, including the specific measurement variable (eg, systolic blood pressure), analysis metric (eg, change from baseline, final value, time to event), method of aggregation (eg, median, proportion), and time point for each outcome. Explanation of the clinical relevance of chosen efficacy and harm outcomes is strongly recommended | 6-7 |
| Participant timeline | #13 | Time schedule of enrolment, interventions (including any run-ins and washouts), assessments, and visits for participants. A schematic diagram is highly recommended (see Figure) | Table 1 |
| Sample size | #14 | Estimated number of participants needed to achieve study objectives and how it was determined, including clinical and statistical assumptions supporting any sample size calculations | 11 |
| Recruitment | #15 | Strategies for achieving adequate participant enrolment to reach target sample size | 11 |
| Allocation: | #16a | Method of generating the allocation sequence (eg, | 8 |

For peer review only

| | | | |
|---|---|---|---|
| sequence generation | | computer-generated random numbers), and list of any factors for stratification. To reduce predictability of a random sequence, details of any planned restriction (eg, blocking) should be provided in a separate document that is unavailable to those who enrol participants or assign interventions | |
| Allocation concealment mechanism | #16b | Mechanism of implementing the allocation sequence (eg, central telephone; sequentially numbered, opaque, sealed envelopes), describing any steps to conceal the sequence until interventions are assigned | 8 |
| Allocation: implementation | #16c | Who will generate the allocation sequence, who will enrol participants, and who will assign participants to interventions | 8 |
| Blinding (masking) | #17a | Who will be blinded after assignment to interventions (eg, trial participants, care providers, outcome assessors, data analysts), and how | 10 |
| Blinding (masking): emergency unblinding | #17b | If blinded, circumstances under which unblinding is permissible, and procedure for revealing a participant's allocated intervention during the trial | n/a |
| Data collection plan | #18a | Plans for assessment and collection of outcome, baseline, and other trial data, including any related processes to promote data quality (eg, duplicate measurements, training of assessors) and a description of study instruments (eg, questionnaires, laboratory tests) along with their reliability and validity, if known. Reference to where data collection forms can be found, if not in the protocol | 9-10 |
| Data collection plan: retention | #18b | Plans to promote participant retention and complete follow-up, including list of any outcome data to be collected for participants who discontinue or deviate from intervention protocols | 10 |
| Data management | #19 | Plans for data entry, coding, security, and storage, including any related processes to promote data | 10 |

For peer review only

| | | | |
|---|---|---|---|
| | | quality (eg, double data entry; range checks for data values). Reference to where details of data management procedures can be found, if not in the protocol | |
| Statistics: outcomes | #20a | Statistical methods for analysing primary and secondary outcomes. Reference to where other details of the statistical analysis plan can be found, if not in the protocol | 11-12 |
| Statistics: additional analyses | #20b | Methods for any additional analyses (eg, subgroup and adjusted analyses) | 12 |
| Statistics: analysis population and missing data | #20c | Definition of analysis population relating to protocol non-adherence (eg, as randomised analysis), and any statistical methods to handle missing data (eg, multiple imputation) | 11 |
| Data monitoring: formal committee | #21a | Composition of data monitoring committee (DMC); summary of its role and reporting structure; statement of whether it is independent from the sponsor and competing interests; and reference to where further details about its charter can be found, if not in the protocol. Alternatively, an explanation of why a DMC is not needed | 12 |
| Data monitoring: interim analysis | #21b | Description of any interim analyses and stopping guidelines, including who will have access to these interim results and make the final decision to terminate the trial | 12 |
| Harms | #22 | Plans for collecting, assessing, reporting, and managing solicited and spontaneously reported adverse events and other unintended effects of trial interventions or trial conduct | 12 |
| Auditing | #23 | Frequency and procedures for auditing trial conduct, if any, and whether the process will be independent from investigators and the sponsor | Audits are randomly performed on trials in the institute (Erasmus MC which is the sponsor) |

For peer review only

| | | | |
|---|---|---|---|
| Research ethics approval | #24 | Plans for seeking research ethics committee / institutional review board (REC / IRB) approval | 12 |
| Protocol amendments | #25 | Plans for communicating important protocol modifications (eg, changes to eligibility criteria, outcomes, analyses) to relevant parties (eg, investigators, REC / IRBs, trial participants, trial registries, journals, regulators) | 12 |
| Consent or assent | #26a | Who will obtain informed consent or assent from potential trial participants or authorised surrogates, and how (see Item 32) | 7-8 |
| Consent or assent: ancillary studies | #26b | Additional consent provisions for collection and use of participant data and biological specimens in ancillary studies, if applicable | 8 |
| Confidentiality | #27 | How personal information about potential and enrolled participants will be collected, shared, and maintained in order to protect confidentiality before, during, and after the trial | 10 |
| Declaration of interests | #28 | Financial and other competing interests for principal investigators for the overall trial and each study site | 14 |
| Data access | #29 | Statement of who will have access to the final trial dataset, and disclosure of contractual agreements that limit such access for investigators | 14 |
| Ancillary and post trial care | #30 | Provisions, if any, for ancillary and post-trial care, and for compensation to those who suffer harm from trial participation | 12 |
| Dissemination policy: trial results | #31a | Plans for investigators and sponsor to communicate trial results to participants, healthcare professionals, the public, and other relevant groups (eg, via publication, reporting in results databases, or other data sharing arrangements), including any publication restrictions | 12 |
| Dissemination | #31b | Authorship eligibility guidelines and any intended | 14 |

For peer review only

| | | | |
|---|---|---|---|
| policy: authorship | | use of professional writers | |
| Dissemination policy: reproducible research | #31c | Plans, if any, for granting public access to the full protocol, participant-level dataset, and statistical code | n/a |
| Informed consent materials | #32 | Model consent form and other related documentation given to participants and authorised surrogates | In Dutch |
| Biological specimens | #33 | Plans for collection, laboratory evaluation, and storage of biological specimens for genetic or molecular analysis in the current trial and for future use in ancillary studies, if applicable | 9 |

The SPIRIT checklist is distributed under the terms of the Creative Commons Attribution License CC-BY-ND 3.0. This checklist can be completed online using https://www.goodreports.org/, a tool made by the EQUATOR Network in collaboration with Penelope.ai

For peer review only

BMJ Open

# The CoDiNOS trial protocol: An international randomized controlled trial of intravenous sildenafil versus inhaled nitric oxide for the treatment of pulmonary hypertension in neonates with congenital diaphragmatic hernia.



| | |
|---|---|
| Journal: | *BMJ Open* |
| Manuscript ID | bmjopen-2019-032122.R2 |
| Article Type: | Protocol |
| Date Submitted by the Author: | 16-Sep-2019 |



| | |
|---|---|
| Complete List of Authors: | Cochius - den Otter, Suzan; Erasmus University Rotterdam, Pediatric Intensive Care<br>Schaible, Thomas; University Medical Center, Mannheim<br>Greenough, Anne; Kings College London, Department of Women and Children's Health, School of Life Sciences, Faculty of Life Science and Medicine<br>van Heijst, Arno; Radboudumc, Division of Neonatology, Department of Pediatrics<br>Patel, Neil; Royal Hospital for Children Glasgow, Departement of Neonatology<br>Allegaert, Karel; Erasmus MC Sophia, Pediatrics, Division of Neonatology<br>van Rosmalen, Joost; Erasmus MC, Biostatistics<br>Tibboel, Dick; Erasmus MC-Sophia Children's Hospital, Intensive Care and Pediatric Surgery |



| | |
|---|---|
| <b>Primary Subject Heading</b>: | Paediatrics |
| Secondary Subject Heading: | Evidence based practice, Intensive care, Pharmacology and therapeutics |
| Keywords: | congenital diaphragmatic hernia, sildenafil, nitric oxide, THERAPEUTICS, pulmonary hypertension |
| | |


SCHOLARONE™
Manuscripts

**The CoDiNOS trial protocol: An international randomized controlled trial of intravenous sildenafil versus inhaled nitric oxide for the treatment of pulmonary hypertension in neonates with congenital diaphragmatic hernia.**

Suzan CM Cochius – den Otter MD[1], Thomas Schaible MD PhD[2], Anne Greenough MD[3], Arno van Heijst MD PhD[4], Neil Patel MD[5], Karel Allegaert MD PhD[1,6], Joost van Rosmalen PhD[7], Dick Tibboel MD PhD[1] on behalf of the CDH EURO Consortium

[1]Intensive Care and department of Pediatric Surgery, Erasmus MC, Rotterdam, the Netherlands. [2]Department of Neonatology Universitätsmedizin Mannheim, Mannheim, Germany. [3] The Asthma UK Centre in Allergic Mechanisms of Asthma; Women and Children's Health, School of Life Course Sciences, Faculty of Life Sciences and Medicine, King's College London, Denmark Hill, London, United Kingdom.[4] Division of Neonatology, Department of Pediatrics, Radboudumc Amalia Children's Hospital, Nijmegen, The Netherlands. [5]Departement of Neonatology, Royal Hospital for Children, Glasgow, United Kingdom. [6]Department of Development and Regeneration, KU-Leuven, Leuven, Belgium. [7]Department of Biostatistics, Erasmus MC, Rotterdam, The Netherlands.

Corresponding author

Suzan CM Cochius – den Otter

Erasmus MC-Sophia Children's Hospital

Intensive Care Unit

Wytemaweg 80

3015 CN Rotterdam

Tel.: (+31) 10 7031769

Fax: (+31) 10 7037162

s.denotter@erasmusmc.nl

**Word count:** 3004

**Keywords:** Congenital Diaphragmatic Hernia, Pulmonary Hypertension, Therapeutics, Sildenafil, Nitric Oxide



For peer review only - http://bmjopen.bmj.com/site/about/guidelines.xhtml

## Abstract

### Introduction

Congenital diaphragmatic hernia (CDH) is a developmental defect of the diaphragm that impairs normal lung development, causing pulmonary hypertension (PH). PH in CDH newborns is the main determinant for morbidity and mortality. Different therapies are still mainly based on "trial and error". Inhaled nitric oxide (iNO) is often the drug of first choice. However, iNO does not seem to improve mortality. Intravenous (iv) sildenafil has reduced mortality in newborns with PH without CDH, but prospective data in CDH patients are lacking.

### Methods and analysis

In an open label, multicenter, international randomized controlled trial in Europe, Canada and Australia, 330 newborns with CDH and PH are recruited over a four-year period (2018-2022). Patients are randomized for iv sildenafil or iNO. Sildenafil is given in a loading dose of 0.4 mg/kg in 3 hours; followed by continuous infusion of 1.6 mg/kg/day, iNO is dosed at 20 ppm.

Primary outcome is absence of PH on day 14 without pulmonary vasodilator therapy and/or absence of death within the first 28 days of life. Secondary outcome measures include clinical and echocardiographic markers of PH in the first year of life.

We hypothesize that sildenafil gives a 25% reduction in the primary outcome from 68% to 48% on day 14, for which a sample size of 330 patients is needed. An intention-to-treat analysis will be performed. A p-value (two-sided) $< 0.05$ is considered significant in all analyses.

### Ethics and dissemination

Ethics approval has been granted by the ethics committee in Rotterdam (MEC-2017-324) and the central Committee on Research Involving Human Subjects (NL60229.078.17) in the Netherlands. The principles of the Declaration of Helsinki, the Medical Research Involving Human Subjects Act, and the national rules and regulations on personal data protection will be used. Parental informed consent will be obtained.

### Registration

Trial registration number NTR6982 (Trial NL6796).




For peer review only - http://bmjopen.bmj.com/site/about/guidelines.xhtml

**Article summary**

**Strengths and limitations of this study**

- **The CoDiNOS trial is the first randomized controlled multicenter trial to evaluate the effect of intravenous sildenafil and compare with iNO on pulmonary hypertension in newborns with CDH.**
- **Treatment allocation is not blinded in the trial. This is not feasible because of variability in iNO equipment and gas mixtures use. Instead, the researchers who analyze the echocardiography to evaluate PH will be blinded to the treatment.**
- **The primary outcome, PH, will be measured using echocardiography instead of just clinical parameters often used in newborns.**
- **There is no non-intervention group, as it is common practice in the centers of the CDH EURO Consortium to give iNO; hence, it is considered unethical to withhold treatment for one group.**
- **Long term follow up of 12 months will give more insight in the course of PH in infants.**

For peer review only



For peer review only - http://bmjopen.bmj.com/site/about/guidelines.xhtml

**Introduction:**

Congenital diaphragmatic hernia (CDH) is a severe developmental defect of the diaphragm with an incidence of approximately 1 in 3000 live births and a mortality of 27% [1]. Because of this defect, the abdominal organs herniate into the chest causing pulmonary hypoplasia and abnormal pulmonary vasculature growth, resulting in pulmonary hypertension (PH) [2]. In adults and children, PH is defined as mean pulmonary artery pressure (mPAP) exceeding 25 mmHg with a pulmonary capillary wedge pressure of minimal 15 mmHg [3].

The normal pulmonary vascular transition of the neonate takes around two months to achieve these low values of mPAP. During fetal life, there is high resistance in the pulmonary circulation which results in most of the blood flow to bypass the lungs through the ductus arteriosus and oval foramen. Immediately after birth, the pulmonary vascular resistance drops and the blood flow to the lungs significantly increases [4]. In contrast, the pulmonary vascular resistance often does not drop adequately in children with CDH due to a decreased vascular bed associated with lung hypoplasia, and an altered development of the pulmonary vasculature with excessive muscularization of the arterioles, with increased thickness of the arterial media and adventitia. Although the presence of lung hypoplasia can be predicted with prenatal parameters, reliable predictors for PH in CDH patients are lacking [5]. The incidence of PH in CDH patients is 68-79% and causes considerable morbidity and mortality [1, 2, 6]. Therapy in newborns with PH , such as inhaled nitric oxide (iNO) and sildenafil, has improved outcomes in general. However, trials in infants with CDH are sparse.

Inhaled nitric oxide (iNO) diffuses rapidly across the alveolar-capillary membrane into the smooth muscle of pulmonary vessels to activate soluble guanylate cyclase. This enzyme mediates many of the biological effects of iNO, and is responsible for the conversion of GTP to cGMP. The increase of intracellular cGMP relaxes smooth muscles via several mechanisms. iNO also causes bronchodilation and has anti-inflammatory and anti-proliferative effects [7]. In term and near term infants with persistent pulmonary hypertension of the newborn (PPHN), iNO decreases the median duration of mechanical ventilation and reduces the need for extracorporeal membrane oxygenation (ECMO). However, in the two available randomized controlled trials (RCT) with a small number of patients with CDH, mortality did not improve and more ECMO treatment was needed despite short-term improved of oxygenation in some treated patients [8, 9]. In the centers of the CDH EURO Consortium, iNO is standard of care in infants with CDH and PH although the positive pharmacodynamic effects in these infants are less convincing then in infants with PPHN [6, 10]. The pathophysiological mechanism of this difference is not understood. In resource poor settings iNO is often unavailable. In



For peer review only - http://bmjopen.bmj.com/site/about/guidelines.xhtml

the search to find another treatment option, trials to evaluate the effect of sildenafil in newborns with PPHN have been conducted [11].

Sildenafil is a selective phosphodiesterase type 5 (PDE5) inhibitor. PDE5 is an enzyme that specifically degrades cGMP. Sildenafil inhibits PDE5, increasing cGMP and NO-mediated vasodilatation of the smooth muscles in arteries. Only five RCTs have been performed in newborns, all non-CDH patients with PPHN. Four of these studies showed a decrease in oxygenation index (OI) and mortality in a setting where iNO was not available, while one trial showed no additional benefit of sildenafil when added to iNO [11]. Although sildenafil is increasingly used in CDH patients, only retrospective data are available [12]. A decrease in pulmonary vascular resistance index and an increase in cardiac output were found in a small group of oral sildenafil-treated infants with CDH refractory to iNO [13]. Intravenous sildenafil improved OI and reversed the right-to-left shunt ratio over the PDA, but it also increased the need for inotropic support [14, 15]. However, its effect on outcome is unknown.

We hypothesize that intravenous sildenafil is superior to iNO. iNO is the therapy of first choice in most centers despite the lack of evidence, and sildenafil is the most promising drug for the treatment of PH in CDH patients and is increasingly being used [6, 12, 16]. However, no studies have been performed comparing iNO with intravenous sildenafil in newborns with CDH and PH or PH alone. Based on the current knowledge, there is equipoise for both treatment modalities.

For peer review only



For peer review only - http://bmjopen.bmj.com/site/about/guidelines.xhtml

**Methods and analysis:**

**Design**

The CoDiNOS trial is a prospective, multicenter, international randomized controlled trial conducted in high volume pediatric surgical centers in Europe, Canada and Australia. The members of the CDH Euro Consortium participating in the trial are listed in the Appendix.

**Objectives**

The primary objective of the study is to determine whether the incidence of PH is lower in CDH patients treated with intravenous sildenafil than in patients treated with iNO, with the primary outcome defined as the absence of PH on echocardiography on day 14 without pulmonary vasodilator therapy and without treatment failure and/or death within the first 28 days after birth. PH is defined as systolic pulmonary arterial pressure> 2/3 systolic systemic pressure and/or right ventricular (RV) dilatation/septal displacement and RV dysfunction +/- left ventricular dysfunction.

The secondary outcomes are:

(1) change in OI after 12 and 24 hours of therapy

(2) overall mortality

(3) the incidence of treatment failure which is defined as:

- inability to maintain preductal saturations above 85% (± 7 kPa or 52 mmHg) or postductal saturations above 70% (±5.3 kPa or 40 mmHg)
- and/or increase in $CO_2$ > 70 mmHg (9.3 kPa) despite optimization of ventilator management
- and/or inadequate oxygen delivery with metabolic acidosis defined as lactate ≥ 5 mmol/l and pH < 7.15 and/or hypotension resistant to fluid therapy and adequate inotropic support resulting in a urine output < 0.5 ml/kg/hour
- and/or lactate ≥ 5 mmol/l and pH < 7.15
- and/or OI consistently ≥ 40

(4) time on intervention drug, defined as intervention drug free days after initiation of the intervention, calculated on day 14

(5) need for ECMO



For peer review only - http://bmjopen.bmj.com/site/about/guidelines.xhtml

(6) ventilator free days on day 28

(7) the use of drugs for PH treatment during the hospital admission

(8) the use of pulmonary and/or cardiac medication at discharge and its total duration of administration

(9) short-term and long-term PH on echocardiography at 24 hours, 28 days/discharge and 6 and 12 months

(10) the incidence of chronic lung disease

(11) the development of neurological abnormalities evaluated with ultrasound of the brain before the start of the trial, after surgery and before discharge

(12) the external validation of the sildenafil PKPD model for the pharmacokinetics and the pharmacodynamic effects of sildenafil

Safety outcomes include adverse events due to the study drugs and the vasoactive-inotropic support score (VIS).

**Patients**

Infants diagnosed with CDH who have PH in the first week after birth, are eligible for the trial if born at or after a gestational age of 34 weeks. The diagnosis of PH is defined as at least two of the following four criteria: (I) systolic pulmonary arterial pressure> 2/3 systolic systemic pressure estimated by echocardiography. (II) RV dilatation/septal displacement, RV dysfunction +/- left ventricular dysfunction. (III) Pre-post ductal $SpO_2$ difference > 10%. (IV) OI >20. Exclusion criteria are a severe chromosomal anomaly which may imply a decision to stop or not to start life-saving medical treatment, severe cardiac anomaly expected to need corrective surgery in the first 60 days of life, renal anomalies associated with oligohydramnios, severe orthopedic and skeletal deformities, which are likely to influence thoracic, and / or lung development and severe anomalies of the central nervous system. Patients who are born in another center and transported with iNO are also excluded from the trial. Patients who received fetal interventions (trachea balloon placement) are not excluded.

Following antenatal diagnosis, the parents are counselled and informed about the study by the clinician or research coordinator. Also, they receive a patient information letter and an informed consent form . If the patient is not born in a participating center or the diagnosis of CDH was not known, parents are counselled after the diagnosis of CDH and are informed about the study. Also,



For peer review only - http://bmjopen.bmj.com/site/about/guidelines.xhtml

they receive written information and an informed consent form. This informed consent form contains consent for the trial and for collection of data and material for future research.

For the development of the protocol the SPIRIT reporting guidelines have been used [17]. This publication is based on protocol version 4, June 13th 2018.

**Patient and public involvement**

Patients and the public were not involved in the development of the trial protocol. However, CDH UK Sparks, as a parent organization, has assessed and commented on the protocol and as provided start-up funding as also mentioned in the funding statement. This organization is and will be regularly informed on progress and results of the trial.

**Study procedures**

*Baseline assessment*

Antenatal ultrasound data about the characteristics of the CDH are collected. These data include the observed/expected lung-head ratio, position of the liver and stomach and the amniotic fluid index. An MRI or an ultrasound is performed depending on local experience and possibilities. If an MRI is performed, the observed/expected fetal lung volume will be calculated. Also data on prenatal interventions are collected. In all mothers, a planned vaginal or caesarean delivery is pursued.

*Randomization, intervention and blinding*

Participants will be randomized using ALEA, which is an online, central randomization service (https://www.aleaclinical.eu). Allocation concealment will be ensured, as the service will not release the randomization code until the patient has been recruited into the trial, which takes place after all baseline characteristics have been added. ALEA randomizes the patient with a computer-generated randomization list, made by the independent statistician of the Data Safety and Monitoring Board. Blocked randomization, with variable block sizes and stratification by center, is used to achieve equal distribution of the two interventions among the participants.

Postnatally, infants are treated according to a standardized protocol for patients with CDH, which is implemented in all participating centers. This protocol was developed with the available evidence and consensus between the participating centers and was updated in June 2016 [10, 16]. If the patient is diagnosed with PH in the first week of life, the patient will be allocated to one of the two study drugs (figure 1). iNO is provided by a tank connected to a ventilator. Different devices are used in different centers. Some centers use integrated systems, making it impossible to disconnect the iNO tank and replace it with another gas to facilitate a blinded intervention. Therefore, the study is



For peer review only - http://bmjopen.bmj.com/site/about/guidelines.xhtml

open label. iNO is given with a starting dose of 20 ppm, which is the maximum dose [18, 19]. Sildenafil is given intravenously, using a loading dose of 0.4mg/kg in 3 hours, followed by continuous infusion of 1.6mg/kg/day [20, 21]. To wean the study drugs a standard protocol is followed (figure 2). The allocated drugs will be restarted as per protocol if criteria for its use are met again before the age of 14 days. To further standardize care, an inotropic support flow chart is included in the study protocol (figure 3). After day 14 treatment of PH will be at the discretion of the local medical team and the study drug can be changed to, for instance, sildenafil orally. The use of bosentan, milrinone and prostin next to the study treatment is allowed. The use of bosentan as add on therapy is allowed and is considered as PH treatment on day 14.The intervention will be prematurely stopped when the patient meets one or more of the defined failure criteria, described in point three of the secondary outcomes. Further treatment will then be at the discretion of the medical team and will be according to the standardized protocol[16]. INO and sildenafil can both be given outside the study protocol. An ECMO-procedure may then be started in centers where ECMO is available. Data of all patients are used in the intention-to-treat analysis.

For peer review only

*Follow up*

After day 14, additional clinical data, such as time on ventilator support (days) and the use of drugs for the treatment of PH, are collected to answer the secondary outcome questions. Also, echocardiographic measurements are taken at 6 and 12 months to evaluate the presence of chronic PH (table 1)

Table 1 Procedures and measurements

| | Day 0-7 before start thera-py | 3 hrs after start silde-nafil | 12hrs after start | 8 am after start | 24hrs after start | Day of surgery, pre-opera-tively | Day after sur-gery | Day of ECMO, pre-cannula-tion | 8 am after start ECMO | Day 14 | Day 28 / before dis-charge | Day 56 | 6 mnth | 12 mnth |
|---|---|---|---|---|---|---|---|---|---|---|---|---|---|---|
| Echocardio -graphy | X | | | | X | | | | | X | X | | X | X |
| Calculation OI | X | | X | | X | | | | | | | | | |
| Calculation VIS score | X | | X | | X | | | | | | | | | |
| Blood sample | X | | | X | | X | X | X | X | X | | | | |
| Tracheal aspirate | X | | | X | | X | X | X | X | X | | | | |
| Urine sample | X | | | X | | X | X | X | X | X | | | | |
| Severity of CLD | | | | | | | | | | | X | X | | |
| Ultrasound | X | | | | | | X | | | | | | | |



For peer review only - http://bmjopen.bmj.com/site/about/guidelines.xhtml

| | | | | | | | | | | | | | | |
|---|---|---|---|---|---|---|---|---|---|---|---|---|---|---|
| brain | | | | | | | | | | | | | | |
| Sildenafil plasma level | | X | | X | | X | X | | X | | | | | |

OI: oxygenation index; VIS score: vasoactive-inotropic support score; CLD: chronic lung disease; ECMO: extracorporeal membrane oxygenation

*Data collection*

Echocardiography parameters are measured by local physicians, centrally collected and reviewed by two blinded independent physicians to reduce inter-observer variation. Demographic and neonatal characteristics as well as data on the clinical course of all patients are entered in a password protected web-based database in Rotterdam (OpenClinica). Upon request the collected data will be available. All centers will keep a logbook of the number of non-participants, including the reasons for not participating. Study documents are securely stored at each study site for 15 years.

*Laboratory testing*

Blood, urine and tracheal samples are collected in most centers during the trial. Blood samples are collected before the start of the study and at different time points until day 14. Some samples will be used to externally validate a NONMEM prediction model for sildenafil. The other samples will be used in future research on biomarkers to predict severity and outcome of PH in CDH patients. The samples are centrifuged for 6 minutes at 3000 rpm [22]. Thereafter, the plasma is removed and stored at –20 degrees Celsius or colder. The total amount of blood taken is maximal 2.5 % of the circulating volume. Blood sampling will only be done if a central or peripheral line is still present and/or in combination with routine laboratory measurements. This way blood sampling is a minimal burden for the patient.

Tracheal aspirate for proteomic analysis is also collected at different time points during routine tracheal suctioning in ventilated patients. Protein profiling with proteomics is used to identify specific groups of proteins that are involved in the pathogenesis of PH. The tracheal aspirates is centrifuged for 6 minutes at 3000 rpm and stored at –80 degrees Celsius [23].

Also, 8-hour urine is collected at different time points. Two samples of 5 ml are taken and stored at –20 degrees Celsius or colder.

*Withdrawal of participants*

Parents may decide to withdraw from the study at any time without any consequences. The investigator can decide to withdraw a patient from the study for urgent medical reasons. In some



For peer review only - http://bmjopen.bmj.com/site/about/guidelines.xhtml

cases, there may be exclusion criteria, which were not known before randomization. If this is the case, the patient will be withdrawn from the study after contacting the study coordinator. With consent of the parents data will still be collected, stored and analyzed to perform an intention-to-treat analysis. These children will be treated according to standard practice [10, 16].

**Sample size calculation**

The sample size calculation is based on a power analysis for the primary outcome, using previously published data on PH. . Lusk et al. showed that PH, defined as >2/3 systemic blood pressure measured on echocardiography, in CDH patients on day 14 has a positive predictive value of 0.8 for death, death or ventilation, and death or ventilator support. PH on day 14 is observed in 64% of CDH patients [24].

Even though the definition of the primary outcome is not the same, we assume a similar outcome percentage of 64% for failing the primary outcome in our trial, the absence of PH on day 14 without pulmonary vasodilator therapy and/or absence of death within the first 28 days of life, in the iNO group. Our aim is to promote practice change, therefor we aim for a clinical significant difference For a 25% relative reduction to 48%, a sample size of 300 patients (150 patients per group) is needed to obtain a power of 80%. This will match a number needed to treat of 6.25. Taking missing data and the effects of correction for covariates into account, we adjust this sample size to 330 patients. In the collaborating centers 550 patients will be born in three years. Based on our earlier trial (VICI trial) we expect to have an inclusion rate of 60%. Therefore, the inclusion of 330 patients should be reached in three years.

**Data analysis**

The patients will be analyzed according to the group they are randomized to (intention-to-treat analysis). A p-value (two-sided) < 0.05 is considered significant in all analyses. The primary endpoint will be analyzed using multiple logistic regression with randomization arm, center, observed/expected head-lung ratio, position of the liver, side of the defect, defect size and ventilation modality as independent variables [25]. If necessary, multiple imputation using the fully conditional specification method will be used to account for missing data in the independent variables. We will perform a sensitivity analyses with adjustment for the use of prostin and milrinone, to account for the effects of these vasodilators on PH.



For peer review only - http://bmjopen.bmj.com/site/about/guidelines.xhtml

The following analyses will be performed for the secondary outcomes. The distribution of VIS score in all study participants will be compared between t=0 and t=12 hours after initiation of drug administration using a Wilcoxon signed rank test. The distribution of changes in OI and VIS score from t=0 to t=12 and t=24 hours will be compared between the randomization groups with a Mann-Whitney test. The overall mortality in the first year of life will be compared between the randomization groups with Kaplan-Meier curves and the log-rank test. The number of treatment failures, the need for ECMO (in ECMO centers), and the need for medication for PH or chronic lung disease at discharge, and during the first year of life, will be compared between randomization groups with chi-square tests. The number of study drug free days at day 14, the number of ventilation-free days until day 28, the fraction of days with need for medical treatment (excluding the study drug) for PH during the hospital admission, and the severity of chronic lung disease using the Bancalari definition, will be compared between randomization groups with Mann-Whitney tests. Deaths will be counted as the worst outcome in these analyses, in accordance with the intention-to-treat principle. The presence of PH at 28 days/discharge, 6 and 12 months according to the echocardiographic parameters will be compared between randomization groups with a chi-square test.

To externally validate the pharmacokinetic model of sildenafil and it active metabolite ( in NONMEM) Normalized prediction distribution errors (NPDE) and Visual Predictive Check (VPC) will be used. Furthermore, the model will be used to predict the drug concentrations from the new data set using simulations, in which we expect that the difference will be less than 20%. To assess whether there is a relationship between the concentration of sildenafil, its active metabolite and the clinical effects, such as OI, VIS score and echocardiography measures, a Mann-Whitney or T test will be used.

**Safety reporting and trial oversight**

All severe adverse events (SAEs) and suspected unexpected serious adverse reactions (SUSARs) are reported from the enrolment until 12 month follow-up. Persistent or significant disability or incapacity that was not expected with the given O/E LHR is evaluated as an SAE. An elective hospital admission is not a SAE. All SAEs and SUSARs are reported to the approving ethics committees in accordance with their requirements. We will report the SAEs and SUSARs that result in death or are life threatening within 7 days of first knowledge. All other SAEs and SUSARs will be reported within a period of maximum 15 days. Once a year throughout the clinical trial, we will submit a safety report to the approving ethics committees and competent authorities of the countries involved.

The trial will be monitored by qualified, independent monitors. The trial is classified as a trial with moderate risk and a specific monitoring plan is in place.



For peer review only - http://bmjopen.bmj.com/site/about/guidelines.xhtml

The data safety monitoring board will monitor the incidence of mortality on a continuous basis. If at some point a large difference in mortality*, defined as an absolute risk increase of 25%,* between the two treatment groups is noticed, the data safety monitoring board may recommend ending the study.

Insurance will cover compensation to patients who suffer harm from trial participation.

**Ethics and dissemination**

Ethics approval has been granted by the local ethics committee in Rotterdam (MEC-2017-324) and by the central Committee on Research Involving Human Subjects (NL60229.078.17) in the Netherlands. The trial will be submitted to the regulatory bodies and the local IRB's in all participating countries. Important amendments will be communicated to all relevant parties. The study will be conducted according to the principles of the Declaration of Helsinki, in accordance with the Medical Research Involving Human Subjects Act, and national rules and regulations on personal data protection. Parental informed consent will be obtained. The results of this study will be disseminated via peer-reviewed publications and implemented in the international guidelines for the treatment of newborns with CDH.

For peer review only



For peer review only - http://bmjopen.bmj.com/site/about/guidelines.xhtml

**Literature**


1. Snoek, K.G., et al., *Conventional Mechanical Ventilation Versus High-frequency Oscillatory Ventilation for Congenital Diaphragmatic Hernia: A Randomized Clinical Trial (The VICI-trial).* Ann Surg, 2015.
2. Robinson, P.D. and D.A. Fitzgerald, *Congenital diaphragmatic hernia.* Paediatr Respir Rev, 2007. **8**(4): p. 323-34; quiz 334-5.
3. Galie, N., et al., *Guidelines for the diagnosis and treatment of pulmonary hypertension: the Task Force for the Diagnosis and Treatment of Pulmonary Hypertension of the European Society of Cardiology (ESC) and the European Respiratory Society (ERS), endorsed by the International Society of Heart and Lung Transplantation (ISHLT).* Eur Heart J, 2009. **30**(20): p. 2493-537.
4. Vali, P. and S. Lakshminrusimha, *The Fetus Can Teach Us: Oxygen and the Pulmonary Vasculature.* Children (Basel), 2017. **4**(8).
5. Russo, F.M., et al., *Lung size and liver herniation predict need for extracorporeal membrane oxygenation but not pulmonary hypertension in isolated congenital diaphragmatic hernia: systematic review and meta-analysis.* Ultrasound Obstet Gynecol, 2017. **49**(6): p. 704-713.
6. Putnam, L.R., et al., *Evaluation of Variability in Inhaled Nitric Oxide Use and Pulmonary Hypertension in Patients With Congenital Diaphragmatic Hernia.* JAMA Pediatr, 2016.
7. Ichinose, F., J.D. Roberts, Jr., and W.M. Zapol, *Inhaled nitric oxide: a selective pulmonary vasodilator: current uses and therapeutic potential.* Circulation, 2004. **109**(25): p. 3106-11.
8. *Inhaled nitric oxide and hypoxic respiratory failure in infants with congenital diaphragmatic hernia. The Neonatal Inhaled Nitric Oxide Study Group (NINOS).* Pediatrics, 1997. **99**(6): p. 838-45.
9. Clark, R.H., et al., *Low-dose nitric oxide therapy for persistent pulmonary hypertension of the newborn. Clinical Inhaled Nitric Oxide Research Group.* N Engl J Med, 2000. **342**(7): p. 469-74.
10. Reiss, I., et al., *Standardized postnatal management of infants with congenital diaphragmatic hernia in Europe: the CDH EURO Consortium consensus.* Neonatology, 2010. **98**(4): p. 354-64.
11. Kelly, L.E., A. Ohlsson, and P.S. Shah, *Sildenafil for pulmonary hypertension in neonates.* Cochrane Database Syst Rev, 2017. **8**: p. CD005494.
12. Lakshminrusimha, S., et al., *Milrinone in congenital diaphragmatic hernia - a randomized pilot trial: study protocol, review of literature and survey of current practices.* Matern Health Neonatol Perinatol, 2017. **3**: p. 27.
13. Noori, S., et al., *Cardiovascular effects of sildenafil in neonates and infants with congenital diaphragmatic hernia and pulmonary hypertension.* Neonatology, 2007. **91**(2): p. 92-100.
14. Bialkowski, A., F. Moenkemeyer, and N. Patel, *Intravenous sildenafil in the management of pulmonary hypertension associated with congenital diaphragmatic hernia.* Eur J Pediatr Surg, 2015. **25**(2): p. 171-6.
15. Kipfmueller, F., et al., *Continuous intravenous sildenafil as an early treatment in neonates with congenital diaphragmatic hernia.* Pediatr Pulmonol, 2018.
16. Snoek, K.G., et al., *Standardized Postnatal Management of Infants with Congenital Diaphragmatic Hernia in Europe: The CDH EURO Consortium Consensus - 2015 Update.* Neonatology, 2016. **110**(1): p. 66-74.
17. Chan, A.W., et al., *SPIRIT 2013 statement: defining standard protocol items for clinical trials.* Ann Intern Med, 2013. **158**(3): p. 200-7.
18. Tworetzky, W., et al., *Inhaled nitric oxide in neonates with persistent pulmonary hypertension.* Lancet, 2001. **357**(9250): p. 118-20.
19. Barrington, K.J., et al., *Nitric oxide for respiratory failure in infants born at or near term.* Cochrane Database Syst Rev, 2017. **1**: p. CD000399.


For peer review only



For peer review only - http://bmjopen.bmj.com/site/about/guidelines.xhtml

20. Steinhorn, R.H., et al., *Intravenous sildenafil in the treatment of neonates with persistent pulmonary hypertension.* J Pediatr, 2009. **155**(6): p. 841-847 e1.
21. Mukherjee, A., et al., *Population pharmacokinetics of sildenafil in term neonates: evidence of rapid maturation of metabolic clearance in the early postnatal period.* Clin Pharmacol Ther, 2009. **85**(1): p. 56-63.
22. Snoek, K.G., et al., *High-Sensitivity Troponin T and N-Terminal Pro-Brain Natriuretic Peptide in Prediction of Outcome in Congenital Diaphragmatic Hernia: Results from a Multicenter, Randomized Controlled Trial.* J Pediatr, 2016. **173**: p. 245-249 e4.
23. Bowler, R.P., M.C. Ellison, and N. Reisdorph, *Proteomics in pulmonary medicine.* Chest, 2006. **130**(2): p. 567-74.
24. Lusk, L.A., et al., *Persistence of pulmonary hypertension by echocardiography predicts short-term outcomes in congenital diaphragmatic hernia.* J Pediatr, 2015. **166**(2): p. 251-6 e1.
25. Lally, K.P., et al., *Standardized reporting for congenital diaphragmatic hernia--an international consensus.* J Pediatr Surg, 2013. **48**(12): p. 2408-15.

**Author contributions**

All investigators of the Consortium described below, have contributed to the design of the trial protocol and have approved this version for submission. Coordinating investigator S Cochius – den Otter and Prof D Tibboel are responsible for all aspects of the study conduct, practically study oversight, recruitment, training of the participating hospitals, reporting of the SAEs and SUSARs, outcome assessment and data management.  Prof D Tibboel, Prof K Allegaert, Dr T Schaible, Dr A van Heijst, Dr A Greenough and Dr N Patel are responsible for study oversight. J van Rosmalen has contributed to statistical methods and will be involved in interpretation of the results. S Cochius- den Otter will lead the dissemination and translation of results with the contribution of all investigators of the CDH EURO Consortium. Also all members will have authority over the data.

**Funding statement**

This work was supported by CDH UK Sparks grant number 16EMC01 and by Stichting Sophia Kinderziekenhuis Fonds grant number S17-19.

**Competing interests**

None

**Data sharing statement:**

Deidentified individual participant data will be made available, in addition to the statistical analysis plan. The data will be made available upon publication to researchers who provide a methodologically sound proposal for use in achieving the goals of the approved proposal. Every center will make data available for sharing after consultation of the PI of that center. Requests can be send to Dick Tibboel (d.tibboel@erasmusmc.nl)

**Figure 1** Trial flow chart



For peer review only - http://bmjopen.bmj.com/site/about/guidelines.xhtml

For peer review only

Flow chart showing the steps of the trial, from birth until 12 months. CDH: congenital diaphragmatic hernia; SAE: serious adverse event; SUSAR: suspected unexpected serious adverse event

**Figure 2** Protocol to wean study drug

Flow chart showing the protocol to wean off inhaled nitric oxide or intravenous sildenafil. iNO: inhaled nitric oxide; ppm: parts per million

**Figure 3** Treatment flow chart of systemic hypotension

Flow chart that is added to the treatment protocol, showing the treatment plan for systemic hypotension. VA ECMO: veno-arterial extracorporeal membrane oxygenation

For peer review only



For peer review only - http://bmjopen.bmj.com/site/about/guidelines.xhtml

Figure 1 Trial flow chart

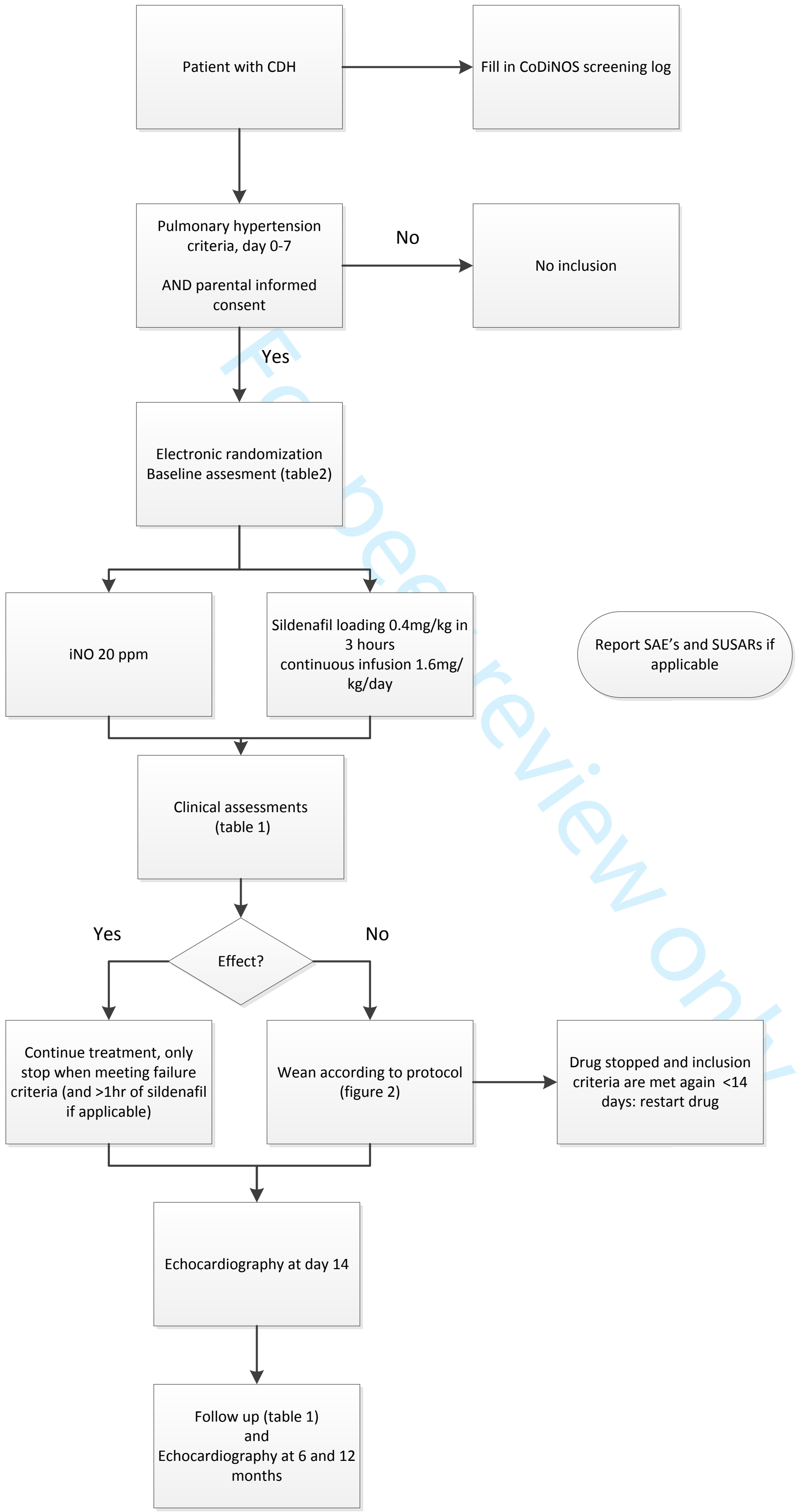


CDH: congenital diaphragmatic hernia; SAE: serious adverse event; SUSAR: suspected unexpected serious adverse event

For peer review only - http://bmjopen.bmj.com/site/about/guidelines.xhtml

Figure 2 Protocol to wean study drug

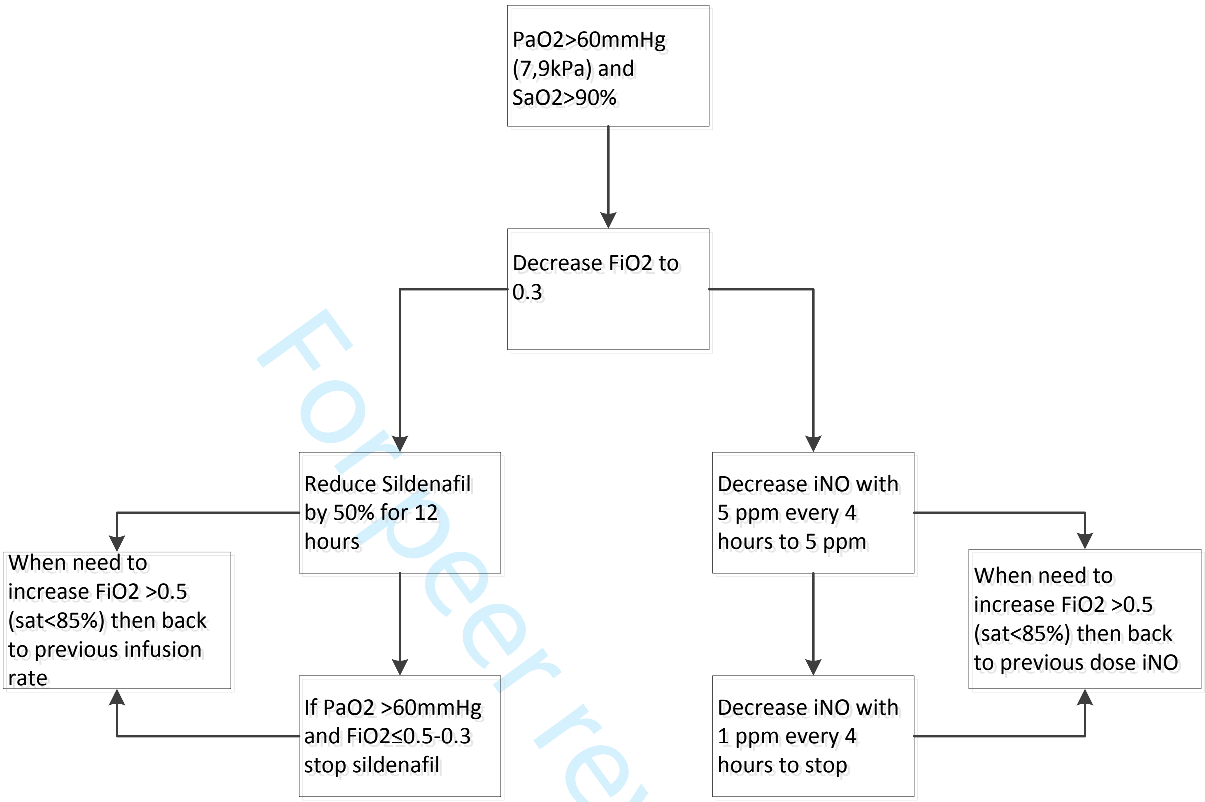

Figure 3: Treatment flow chart of systemic hypotension

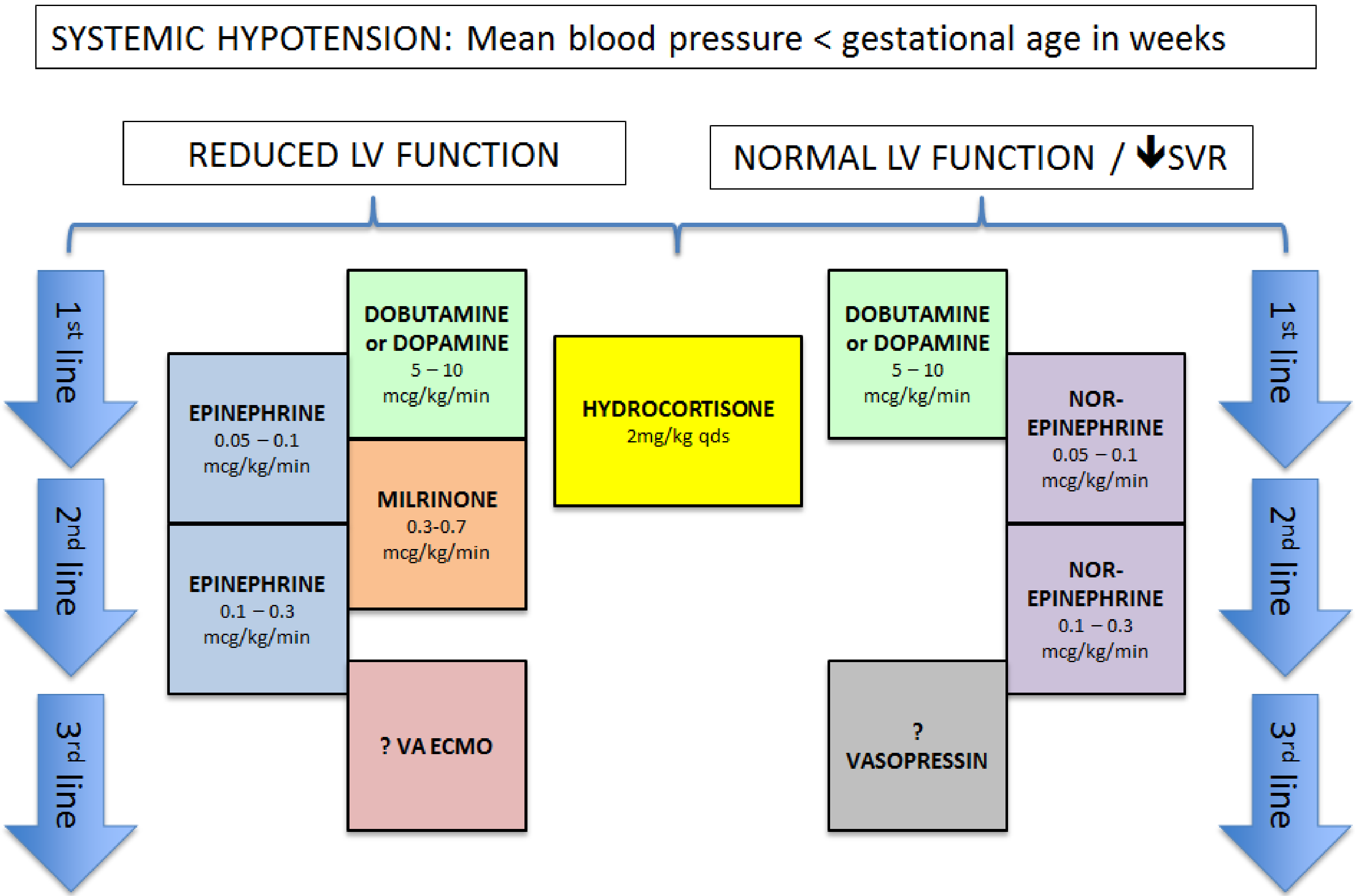

**Appendix:**

CDH Euro Consortium:

Germany: Florian Kipfmueller, Department of Neonatology and Pediatric Critical Care Medicine, University Children's Hospital, Bonn. Spain: Maria Dolores Elorza, Ana Sanchez, Neonatology Department, Leopoldo Martinez, Pediatric Surgery Department, Carlos Labrandero, Viviana Arreo, Pediatric Cardiology Division, Hospital Universitario La Paz, Madrid. Africa Pertierra Cortada, Jordi Clotet Caba Neonatology Department, Hospital Sant Joan de Déu, Barcelona. Marta Aguar, Ana Gimeno, Raquel Escrig, Division of Neonatology, University and Polytechnic Hospital La Fe Valencia. Italy: Irma Capolupo, Pietro Bagolan, Department of Medical and Surgical Neonatology, Bambino Gesu' Children's Hospital, Rome. Fabrizio Ciralli, Genny Raffaeli, Giacomo Cavallaro, Valentina Condò, Fondazione IRCCS Ca' Granda Ospedale Maggiore Policlinico, NICU, University of Milan, Department of Clinical Sciences and Community Health. United Kingdom: United Kingdom - Paul D. Losty Department Of Paediatric Surgery, Division of Child Health, Alder Hey Children's Hospital NHS Foundation Trust, University of Liverpool , Marie Horan, Paediatric Intensive Care Alder Hey Children's Hospital NHS Foundation Trust, University of Liverpool. Nimish V. Subhedar, NICU, Liverpool Women's Hospital, Liverpool. Yogen Singh, Department of Neonatology, Cambridge University Hospitals NHS Foundation trust, Cambridge. Emma E. Williams, The Asthma UK Centre in Allergic Mechanisms of Asthma; Women and Children's Health, School of Life Course Sciences, Faculty of Life Sciences and Medicine, King's College London, Denmark Hill, London. Theodore Dassios, Ravindra Bhat, King's College Hospital NHS Foundation Trust, London. Austria: Jennifer B. Brandt, Alexandra Kreissl, Angelika Berger, Department of Pediatrics and Adolescent Medicine, Division of Neonatology, Pediatric Intensive Care Medicine and Neuropediatrics, Medical University of Vienna. Berndt Urlesberger, Division of Neonatology, Department of Pediatrics and Adolescent Medicine, Medical University of Graz. Sweden: Carmen Mesas Burgos, Björn Frenckner, Department of Pediatric Surgery, Björn Larrson, Pediatric Intensive Care Unit, Karolinska University Hospital, Stockholm. Portugal: Carla Pinto, Serviço de Cuidados Intensivos Pediátricos, Hospital Pediátrico, Centro Hospitalar e Universitário de Coimbra, Coimbra. Joana Saldaha, Department of Neonatology, Hospital de Santa Maria, Lisbon. Belgium: Anne Debeer, Anne Smits, Neonatology, University Hospitals Leuven, Leuven. Norway: Ragnhild Emblem, Department of Pediatric Surgery, Oslo University Hospital, Oslo. Canada: Richard Keijzer, Department of Surgery, Yassar Elsayed, Department of Neonatology, Pediatrics and Child Health, University of Manitoba and Children's Hospital Research Institute of Manitoba. Australia: David Tingay, Department of Neonatology, Royal Children's Hospital, Melbourne, Australia. The Netherlands: Ulrike Kraemer, Intensive Care and department of Pediatric Surgery, Erasmus MC, Rotterdam.

For peer review only

# Reporting checklist for protocol of a clinical trial.

Based on the SPIRIT guidelines.

## Instructions to authors

Complete this checklist by entering the page numbers from your manuscript where readers will find each of the items listed below.

Your article may not currently address all the items on the checklist. Please modify your text to include the missing information. If you are certain that an item does not apply, please write "n/a" and provide a short explanation.

Upload your completed checklist as an extra file when you submit to a journal.

In your methods section, say that you used the SPIRIT reporting guidelines, and cite them as:

Chan A-W, Tetzlaff JM, Altman DG, Laupacis A, Gøtzsche PC, Krleža-Jerić K, Hróbjartsson A, Mann H, Dickersin K, Berlin J, Doré C, Parulekar W, Summerskill W, Groves T, Schulz K, Sox H, Rockhold FW, Rennie D, Moher D. SPIRIT 2013 Statement: Defining standard protocol items for clinical trials. Ann Intern Med. 2013;158(3):200-207

For peer review only

| | | Reporting Item | Page Number |
|---|---|---|---|
| Title | #1 | Descriptive title identifying the study design, population, interventions, and, if applicable, trial acronym | 1 |
| Trial registration | #2a | Trial identifier and registry name. If not yet registered, name of intended registry | 2 |
| Trial registration: data set | #2b | All items from the World Health Organization Trial Registration Data Set | 8 |
| Protocol version | #3 | Date and version identifier | 8 |
| Funding | #4 | Sources and types of financial, material, and other support | 14 |
| Roles and responsibilities: contributorship | #5a | Names, affiliations, and roles of protocol contributors | 14 and appendix |
| Roles and | #5b | Name and contact information for the trial sponsor | 1 |

| | | | |
|---|---|---|---|
| responsibilities: sponsor contact information | | | |
| Roles and responsibilities: sponsor and funder | #5c | Role of study sponsor and funders, if any, in study design; collection, management, analysis, and interpretation of data; writing of the report; and the decision to submit the report for publication, including whether they will have ultimate authority over any of these activities | 14 |
| Roles and responsibilities: committees | #5d | Composition, roles, and responsibilities of the coordinating centre, steering committee, endpoint adjudication committee, data management team, and other individuals or groups overseeing the trial, if applicable (see Item 21a for data monitoring committee) | 14 |
| Background and rationale | #6a | Description of research question and justification for undertaking the trial, including summary of relevant studies (published and unpublished) examining benefits and harms for each intervention | 4-5 |
| Background and rationale: choice of comparators | #6b | Explanation for choice of comparators | 4-5 |
| Objectives | #7 | Specific objectives or hypotheses | 6 |
| Trial design | #8 | Description of trial design including type of trial (eg, parallel group, crossover, factorial, single group), allocation ratio, and framework (eg, superiority, equivalence, non-inferiority, exploratory) | 6 |
| Study setting | #9 | Description of study settings (eg, community clinic, academic hospital) and list of countries where data will be collected. Reference to where list of study sites can be obtained | 6, 14, appendix |
| Eligibility criteria | #10 | Inclusion and exclusion criteria for participants. If applicable, eligibility criteria for study centres and | 7 |

For peer review only

| | | | |
|---|---|---|---|
| | | individuals who will perform the interventions (eg, surgeons, psychotherapists) | |
| Interventions: description | #11a | Interventions for each group with sufficient detail to allow replication, including how and when they will be administered | 8 |
| Interventions: modifications | #11b | Criteria for discontinuing or modifying allocated interventions for a given trial participant (eg, drug dose change in response to harms, participant request, or improving / worsening disease) | 9 |
| Interventions: adherance | #11c | Strategies to improve adherence to intervention protocols, and any procedures for monitoring adherence (eg, drug tablet return; laboratory tests) | n/a |
| Interventions: concomitant care | #11d | Relevant concomitant care and interventions that are permitted or prohibited during the trial | 8 |
| Outcomes | #12 | Primary, secondary, and other outcomes, including the specific measurement variable (eg, systolic blood pressure), analysis metric (eg, change from baseline, final value, time to event), method of aggregation (eg, median, proportion), and time point for each outcome. Explanation of the clinical relevance of chosen efficacy and harm outcomes is strongly recommended | 6-7 |
| Participant timeline | #13 | Time schedule of enrolment, interventions (including any run-ins and washouts), assessments, and visits for participants. A schematic diagram is highly recommended (see Figure) | Table 1 |
| Sample size | #14 | Estimated number of participants needed to achieve study objectives and how it was determined, including clinical and statistical assumptions supporting any sample size calculations | 11 |
| Recruitment | #15 | Strategies for achieving adequate participant enrolment to reach target sample size | 11 |
| Allocation: | #16a | Method of generating the allocation sequence (eg, | 8 |

For peer review only

| | | | |
|---|---|---|---|
| sequence generation | | computer-generated random numbers), and list of any factors for stratification. To reduce predictability of a random sequence, details of any planned restriction (eg, blocking) should be provided in a separate document that is unavailable to those who enrol participants or assign interventions | |
| Allocation concealment mechanism | #16b | Mechanism of implementing the allocation sequence (eg, central telephone; sequentially numbered, opaque, sealed envelopes), describing any steps to conceal the sequence until interventions are assigned | 8 |
| Allocation: implementation | #16c | Who will generate the allocation sequence, who will enrol participants, and who will assign participants to interventions | 8 |
| Blinding (masking) | #17a | Who will be blinded after assignment to interventions (eg, trial participants, care providers, outcome assessors, data analysts), and how | 10 |
| Blinding (masking): emergency unblinding | #17b | If blinded, circumstances under which unblinding is permissible, and procedure for revealing a participant's allocated intervention during the trial | n/a |
| Data collection plan | #18a | Plans for assessment and collection of outcome, baseline, and other trial data, including any related processes to promote data quality (eg, duplicate measurements, training of assessors) and a description of study instruments (eg, questionnaires, laboratory tests) along with their reliability and validity, if known. Reference to where data collection forms can be found, if not in the protocol | 9-10 |
| Data collection plan: retention | #18b | Plans to promote participant retention and complete follow-up, including list of any outcome data to be collected for participants who discontinue or deviate from intervention protocols | 10 |
| Data management | #19 | Plans for data entry, coding, security, and storage, including any related processes to promote data | 10 |

For peer review only

| | | | |
|---|---|---|---|
| | | quality (eg, double data entry; range checks for data values). Reference to where details of data management procedures can be found, if not in the protocol | |
| Statistics: outcomes | #20a | Statistical methods for analysing primary and secondary outcomes. Reference to where other details of the statistical analysis plan can be found, if not in the protocol | 11-12 |
| Statistics: additional analyses | #20b | Methods for any additional analyses (eg, subgroup and adjusted analyses) | 12 |
| Statistics: analysis population and missing data | #20c | Definition of analysis population relating to protocol non-adherence (eg, as randomised analysis), and any statistical methods to handle missing data (eg, multiple imputation) | 11 |
| Data monitoring: formal committee | #21a | Composition of data monitoring committee (DMC); summary of its role and reporting structure; statement of whether it is independent from the sponsor and competing interests; and reference to where further details about its charter can be found, if not in the protocol. Alternatively, an explanation of why a DMC is not needed | 12 |
| Data monitoring: interim analysis | #21b | Description of any interim analyses and stopping guidelines, including who will have access to these interim results and make the final decision to terminate the trial | 12 |
| Harms | #22 | Plans for collecting, assessing, reporting, and managing solicited and spontaneously reported adverse events and other unintended effects of trial interventions or trial conduct | 12 |
| Auditing | #23 | Frequency and procedures for auditing trial conduct, if any, and whether the process will be independent from investigators and the sponsor | Audits are randomly performed on trials in the institute (Erasmus MC which is the sponsor) |

For peer review only

| | | | |
|---|---|---|---|
| Research ethics approval | #24 | Plans for seeking research ethics committee / institutional review board (REC / IRB) approval | 12 |
| Protocol amendments | #25 | Plans for communicating important protocol modifications (eg, changes to eligibility criteria, outcomes, analyses) to relevant parties (eg, investigators, REC / IRBs, trial participants, trial registries, journals, regulators) | 12 |
| Consent or assent | #26a | Who will obtain informed consent or assent from potential trial participants or authorised surrogates, and how (see Item 32) | 7-8 |
| Consent or assent: ancillary studies | #26b | Additional consent provisions for collection and use of participant data and biological specimens in ancillary studies, if applicable | 8 |
| Confidentiality | #27 | How personal information about potential and enrolled participants will be collected, shared, and maintained in order to protect confidentiality before, during, and after the trial | 10 |
| Declaration of interests | #28 | Financial and other competing interests for principal investigators for the overall trial and each study site | 14 |
| Data access | #29 | Statement of who will have access to the final trial dataset, and disclosure of contractual agreements that limit such access for investigators | 14 |
| Ancillary and post trial care | #30 | Provisions, if any, for ancillary and post-trial care, and for compensation to those who suffer harm from trial participation | 12 |
| Dissemination policy: trial results | #31a | Plans for investigators and sponsor to communicate trial results to participants, healthcare professionals, the public, and other relevant groups (eg, via publication, reporting in results databases, or other data sharing arrangements), including any publication restrictions | 12 |
| Dissemination | #31b | Authorship eligibility guidelines and any intended | 14 |

For peer review only

| | | | |
|---|---|---|---|
| policy: authorship | | use of professional writers | |
| Dissemination policy: reproducible research | #31c | Plans, if any, for granting public access to the full protocol, participant-level dataset, and statistical code | n/a |
| Informed consent materials | #32 | Model consent form and other related documentation given to participants and authorised surrogates | In Dutch |
| Biological specimens | #33 | Plans for collection, laboratory evaluation, and storage of biological specimens for genetic or molecular analysis in the current trial and for future use in ancillary studies, if applicable | 9 |

The SPIRIT checklist is distributed under the terms of the Creative Commons Attribution License CC-BY-ND 3.0. This checklist can be completed online using https://www.goodreports.org/, a tool made by the EQUATOR Network in collaboration with Penelope.ai

For peer review only
